# Ladder-type π-conjugated frameworks with multi-heteroatom modulation for narrowband violet-blue multiple-resonance emitters with a low CIE_*y*_ of 0.03

**DOI:** 10.1039/d5sc09014h

**Published:** 2026-01-26

**Authors:** Jian-Rong Wu, Yue-Jian Yang, Hai-Tian Yuan, Shi-Jie Ge, Yang-Kun Qu, Hai-Xiao Jiang, Yin Liu, Dong-Ying Zhou, Liang-Sheng Liao, Zuo-Quan Jiang

**Affiliations:** a State Key Laboratory of Bioinspired Interfacial Materials Science, Institute of Functional Nano & Soft Materials (FUNSOM), Soochow University Suzhou 215123 Jiangsu PR China zqjiang@suda.edu.cn; b Macao Institute of Materials Science and Engineering, Macau University of Science and Technology Taipa Macau 999078 China

## Abstract

The development of violet-blue organic light-emitting diodes (OLEDs) with narrowband emission, high color purity and efficiency remains a formidable challenge, particularly under the stringent requirements of the wide-color-gamut display standards. Here, we propose a molecular design strategy that integrates oxygen-bridged triarylboron π-extension with precise heteroatom modulation to construct tetraboron-based MR-TADF emitters 4M-BOB4 and 4F-BOB4. The emitters adopt a ladder-type fused framework with a highly ordered arrangement of boron, nitrogen, and oxygen atoms, while fluorine substitution shifts the emission region from deep-blue to violet-blue. The incorporation of oxygen atoms not only regulates electronic delocalization and frontier orbital distribution, but also contributes to sharpening the emission bandwidth through localized conjugation modulation. As a result, 4F-BOB4 exhibits ultra-narrow emission in solution (with a full-width at half maximum of 14 nm), and corresponding OLED devices exhibit a maximum external quantum efficiency (EQE_max_) of 20.9%. Notably, the device based on 4F-BOB4 exhibits a CIE_*y*_ value ≤0.030, underscoring its status as one of the most color-pure violet-blue emitters reported to date. This work highlights the potential of rational π-framework engineering and localized electronic modulation as a generalizable approach to next-generation high-performance narrowband OLEDs.

## Abstract 2

A ladder-type π-conjugated framework with multi-heteroatom modulation was designed, enabling 4F-BOB4 to serve as an ultra-narrowband violet-blue multi-resonance emitter with a full width at half maximum of 14 nm and a low CIE*_y_* of 0.03 in OLED device.

## Graphical abstract

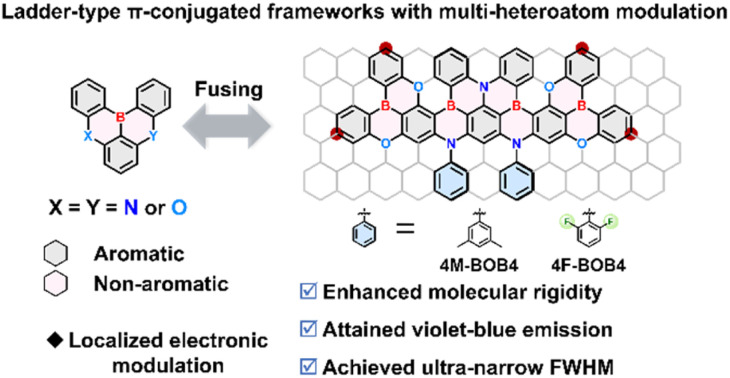

## Introduction

Achieving narrowband emission is crucial for fulfilling the wide color gamut requirements in the development of organic light-emitting diodes (OLEDs).^1–3^ Thermally activated delayed fluorescence (TADF) emitters offer a pathway to high efficiency, but often suffer from spectral broadening that compromises color purity.^4–12^ Multi-resonance TADF (MR-TADF) materials overcome this limitation by embedding heteroatoms with opposing electronic effects into polycyclic aromatic frameworks, generating localized multi-resonance interactions.^13–24^ This approach precisely modulated the spatial separation of the highest occupied molecular orbital (HOMO) and the lowest unoccupied molecular orbital (LUMO) at the atomic level, effectively minimizing the singlet-triplet energy gap (Δ*E*_ST_) between the singlet (S_1_) and triplet (T_1_) excited states. Such a configuration not only suppresses molecular vibrational relaxation but also affords narrowband emission with a small full width at half maximum (FWHM) and a rapid radiative decay rate.^25,26^ Leveraging these advantages, MR-TADF frameworks have been successfully extended to span the visible spectrum and have attracted considerable attention in both academic and industrial fields.^27–32^

**Fig. 1:**
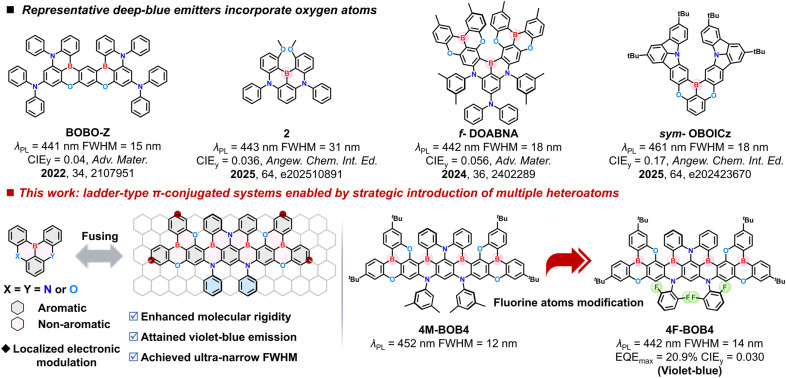
Molecular design strategy for deep-blue emission *via* oxygen-atom functionalization and representative chemical structures.

Despite significant progress, achieving highly saturated deep-blue emission remains one of the most persistent challenges in organic optoelectronics, primarily because the color purity of blue emitters is extremely sensitive to both spectral broadening and residual long-wavelength tails.^3,33–44^ In particular, achieving CIE_*y*_ ≤ 0.046 is widely regarded as a “holy grail” in display technology, driving continuous innovation in both materials design and device engineering.^44–52^ Early MR-TADF emitters, such as DABNA-1 (ref. 14) and *ν*-DABNA,^31^ exhibited high color purity with emission maxima at 459 and 469 nm (FWHM = 18 and 28 nm, respectively) and promising external quantum efficiencies (EQEs). Subsequent approaches, including the introduction of oxygen atoms into B/N-based frameworks (Fig. 1),^46,53–57^ enabled hypsochromic shifts while maintaining short-range charge-transfer (SRCT) characteristics, producing blue-emitting materials with CIE_*y*_ values below 0.10. Furthermore, 5,9-dioxa-13*b*-boranaphtho[3,2,1-*de*]anthracene (DOBNA)^58^ features one of the highest S_1_ energy levels among MR-TADF scaffolds, allowing violet-blue emission to be maintained even in extended conjugated systems.^59,60^ Building upon this, Hatakeyama and Zysman-Colman/Adachi groups independently designed and synthesized novel extended heterohelicene structures (*f*-DOABNA^61^ and DOB2-DABNA-A-NP^62^) by fusing DABNA-1 and DOBNA fragments. Zhang *et al.* also developed a deep-blue emitter, *t*BO-4B, through a linearly fully fused acceptor–donor–acceptor molecular design incorporating the DOBNA unit.^47^ These materials exhibited deep-blue emissions, along with a CIE_*y*_ value of 0.04. Notably, emitters achieving CIE_*y*_ ≤ 0.03 are exceedingly rare and are often classified as ultra-deep-blue or even near-UV systems due to their proximity to the lower vertex of the CIE 1931 chromaticity diagram.^63,64^ Meanwhile, Hattori *et al.* developed a series of *N*,*N*-bridged triphenylboranes featuring methoxy substituents *ortho* to the boron center, which allow fine-tuning of the HOMO–LUMO gap and consequently yield spectrally narrow and blue-shifted emission.^65^ Their relatively large Δ*E*_ST_ and the low reverse intersystem crossing (RISC) rate constrained their overall device performance. Theoretical and experimental studies have since demonstrated that rigidifying the molecular framework through π-conjugation extension with the MR effect can extend span distribution of SRCT within molecules, thereby reducing Δ*E*_ST_, increasing oscillator strength (*f*_osc_) and promoting a fast RISC process; striking an optimal balance between the conjugation-driven red shift and bandwidth narrowing is critical.^66–71^ Therefore, the development of molecular frameworks capable of delivering deep-blue emission with even lower CIE_*y*_ values while retaining high efficiency and narrow spectral width represents a compelling scientific challenge and holds considerable technological significance for next-generation ultra-high-definition displays.

In this work, we propose a molecular design strategy that introduces multiple heteroatoms to enable stepwise π-extension *via* ladder-type fusion, yielding a tetraboron-based deep-blue emitter, 4M-BOB4. Building on this ladder-type architecture, a fluorinated analogue, 4F-BOB4, was developed by integrating electron-withdrawing fluorine substituents to further fine-tune the electronic structure and induce a hypsochromic shift. Both emitters feature rigid ladder-type π-conjugated frameworks embedding boron, nitrogen, oxygen, and fluorine atoms in a highly ordered fashion, where BN units form the MR core and oxygen atoms modulate local conjugation to enhance color purity. 4F-BOB4 pushes the emission further into the violet-blue region and displays an ultra-narrow emission in solution with a FWHM of 14 nm. Notably, the 4F-BOB4-based device delivers a peak at 443 nm with an even narrower FWHM of 17 nm, an EQE_max_ of 20.9%, and an exceptionally low CIE_*y*_ of 0.030. Such ultralow-y emission not only represents an intrinsically purer blue with substantially suppressed green components, but also enables color coordinates that can meet or surpass the BT.2020 standard, thereby expanding the attainable display color gamut. These findings underscore the effectiveness of rational π-framework construction and localized electronic modulation in achieving high-performance violet-blue MR-TADF emitters, providing a generalizable design principle for future narrowband OLEDs toward ultra-high-definition display applications.

## Results and discussion

**Fig. 2:**
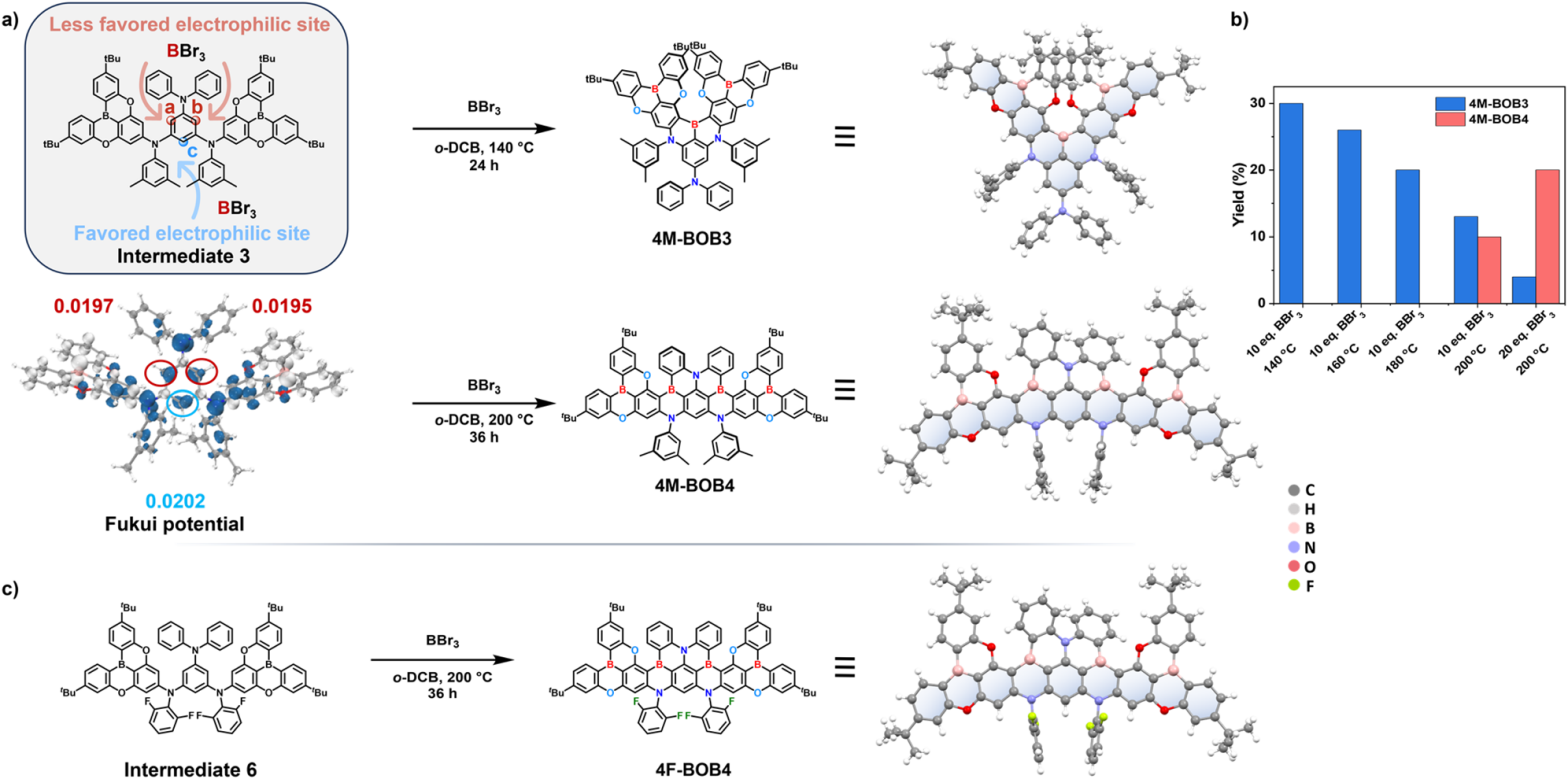
(a) Electrophilic borylation reactions affording 4M-BOB3 and 4M-BOB4 and their crystal structures; (b) reaction conditions and isolated yields; and (c) synthesis and crystal structure of 4F-BOB4.

The ladder-type MR-TADF emitters 4M-BOB4 and 4F-BOB4 are illustrated in Fig. 1, and their corresponding synthetic routes are summarized in Scheme S1 (SI). In our design, the DOBNA fragment was pre-installed to enable stepwise boron incorporation into the π-framework. The key intermediates 3 and 6 were assembled *via* sequential Buchwald–Hartwig couplings. Intriguingly, in contrast to previous reports,^61,62^ a one-pot high-temperature borylation with excess BBr_3_ afforded an unexpected non-helical double-borylated product, which facilitated the successful construction of the ladder-type tetraboron MR-TADF framework, 4M-BOB4 (Fig. 2a). Its structure closely resembles that of *t*BO-4B reported by Zhang *et al.*,^47^ although the possibility of such isomerism was not considered in their study. Regioselectivity investigations revealed a temperature- and stoichiometry-dependent transformation: treatment of intermediate 3 with 10 equiv. BBr_3_ at 140 °C produced 4M-BOB3, consistent with literature outcomes,^61,62^ whereas elevating the temperature to 200 °C afforded 4M-BOB4 in 10% yield. Increasing the BBr_3_ amount to 20 equiv. enhanced the yield to 20%, confirming the critical role of boron concentration and fusion thermodynamics in directing the reaction pathway (Fig. 2b). Strategic fluorination in this reaction allows precise electronic modulation and induces a hypsochromic emission shift, affording the formation of the violet-blue emitter 4F-BOB4 (Fig. 2c). The molecular structures of 4M-BOB3, 4M-BOB4 and 4F-BOB4 were unambiguously verified by using ^1^H and ^13^C nuclear magnetic resonance (NMR) spectra (Fig. S1–S16) and MALDI-TOF-MS (Fig. S17), and their structures by X-ray crystallography (for detailed information, see Fig. S18–S20 and Tables S1–S3). Single-crystal X-ray analysis reveals that the DOBNA unit in the helical conformation of 4M-BOB3 adopts a larger dihedral angle. In contrast, the ladder-type frameworks of 4M-BOB4 and 4F-BOB4 exhibit enhanced planar rigidity. Furthermore, structural features such as the large torsion angles (80.4–88.1°) of the peripheral *m*-xylene and *m*-difluorobenzene units, together with the *tert*-butyl groups located at the meta positions relative to the oxygen atoms, effectively suppress intermolecular packing, and no significant π–π interactions are observed (Fig. S21–S24). Thermal robustness was further confirmed by thermogravimetric analysis (TGA), which revealed high decomposition temperatures (*T*_d_, corresponding to 5% weight loss) of 503 and 508 °C for 4M-BOB4 and 4F-BOB4, respectively (Fig. S25). To evaluate the energy level of the MR-TADF emitters, the cyclic voltammetry (CV) measurements were performed to measure the oxidation potentials. The HOMO energy levels were estimated to be −5.21 and −5.36 eV, respectively, while the corresponding LUMO energy levels of 4M-BOB4 and 4F-BOB4 were found to be −2.47 and −2.57 eV from optical bandgaps (*E*_g_) and HOMO levels (Fig. S26 and Table S4).

**Fig. 3:**
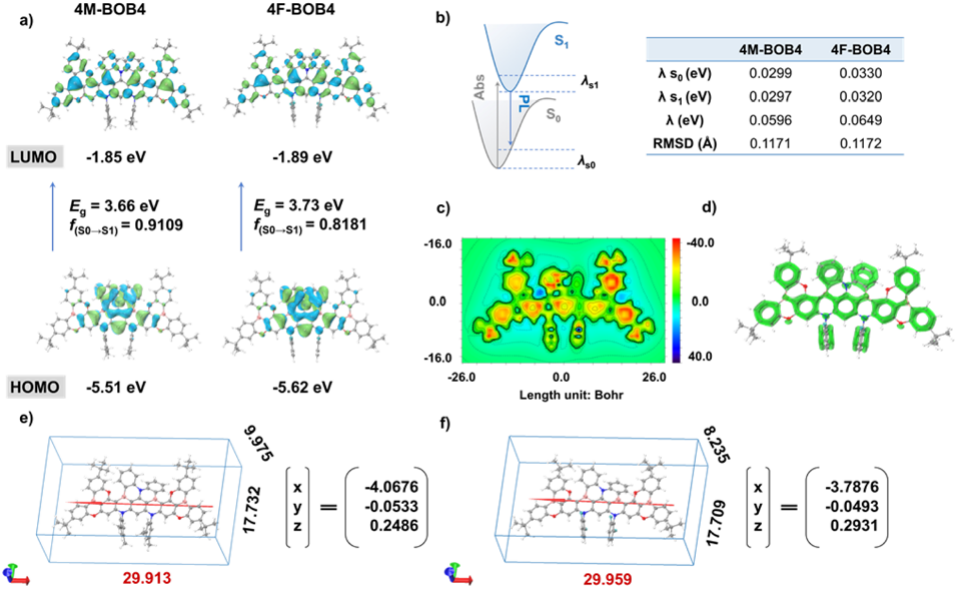
(a) The HOMO and LUMO distributions and energy level diagrams of 4M-BOB4 and 4F-BOB4, respectively; (b) the calculated reorganization energy (*λ*) and root-mean-square-deviation values of 4M-BOB4 and 4F-BOB4, respectively; (c) 2D ICSS map of 4M-BOB4 at 1 Å above the *XY* plane; (d) LOL-π analysis of the luminescent core; the calculated molecular geometries and transition dipole moments of (e) 4M-BOB4 and (f) 4F-BOB4.

To elucidate the structural origin and formation mechanism of the ladder-type conformation, the electronic and structural properties of intermediates 3 and 6 (Fig. 2) were investigated using density functional theory (DFT) and time-dependent DFT (TD-DFT) at the PBE0/def2-SVP level of theory. The HOMO distribution and electrostatic potential (ESP) maps of intermediates 3 and 6 revealed that the active sites a, b and c exhibit relatively lower electrostatic potentials and higher electron cloud densities, thereby favoring electrophilic borylation (Fig. S27 and S28). Furthermore, the orbital-weighted Fukui function analysis^72,73^ identified site c on the central benzene ring of intermediate 3 as the most reactive position, displaying a slightly higher *f*-value (0.0202) compared to sites a (0.0197) and b (0.0195). Consistent with these findings, under milder conditions (140 °C), the borylated product 4M-BOB3 was obtained, analogous to previously reported *f*-DOABNA and DOB2-DABNA-A-NP structures. The frontier molecular orbital (FMO) distributions illustrate that the HOMO and LUMO are localized over distinct atomic regions, highlighting the spatial separation characteristics of MR systems (Fig. 3a). Correspondingly, the hole and electron analyses of the excited states confirmed the SRCT nature of these emitters (Fig. S29–S31). For 4F-BOB4, the incorporation of the electronegative fluorine atoms at the *ortho* position relative to the nitrogen atom enhances the electron-withdrawing character of the molecule, as evidenced by ESP mapping (Fig. S32). Such localized electronic modulation reduces the overall electron density of the π-conjugated framework, preferentially stabilizing the HOMO while exerting minimal influence on the LUMO. Consequently, the optical bandgap widens, leading to a pronounced hypsochromic shift compared with 4M-BOB4.

The structural relaxation accompanying excitation was examined through vibrational analysis to investigate the effect of the ladder-type fused framework on spectral narrowing. 4M-BOB4 exhibits a significantly smaller root-mean-square deviation (RMSD) between ground (S_0_) and S_1_ states (0.1171 Å) than 4M-BOB3 (0.2829 Å), indicating suppressed vibrational distortion upon excitation and thereby contributing to its narrowband emission (Fig. S33 and S34). Furthermore, the reorganization energy (*λ*) and Huang–Rhys (HR) factors were calculated to quantify the extent of conformational changes caused by electronic transitions (Fig. 3b and S35). 4M-BOB4 and 4F-BOB4 exhibit notably lower reorganization energies (0.0596 and 0.0649 eV, respectively) compared with 4M-BOB3 (0.1098 eV), along with small HR factors, theoretically supporting their high-efficiency and ultra-narrowband emission characteristics. In the high-frequency region (>500 cm^−1^), the rigid ladder-type backbone effectively suppresses stretching vibrations, thereby diminishing shoulder peak intensity in the emission spectrum (Fig. S36–S38). In contrast, low-frequency rocking modes associated with *tert*-butyl and methyl groups dominate, which favor the maintenance of narrowband emission by minimizing vibronic coupling.

The nucleus-independent chemical shift (NICS) and two-dimensional isochemical shielding surface (2D-ICSS) analyses were performed to elucidate the aromaticity pattern of 4M-BOB4 and 4F-BOB4. The weak aromatic character is evidenced by their small NICS(1)_*ZZ*_ values (−1.8 to −2.8 ppm) for the heterocycles and the deshielded regions observed in the ICSS plots (Fig. 3c, S39 and S40). Such localized aromaticity contributes to the suppression of spectral shoulder peaks, thereby promoting narrowband emission. Meanwhile, the localized π-conjugation disruption mitigates red-shifting and enables precise tuning of the emission profile.^74–78^ Furthermore, localized orbital locator for π electrons (LOL-π) analysis further reveals extensive π-electron delocalization across the molecular backbone, facilitating efficient charge transport and enhancing molecular stability. Notably, a slight interruption of LOL-π density near the oxygen atom can be attributed to the lone-pair electrons and high electronegativity of the heteroatom,^54^ which locally restrict π-electron delocalization (Fig. 3d). The transition dipole moment (TDM) simulations for 4M-BOB4 and 4F-BOB4 (Fig. 3e and f) reveal that their transition dipole directions are aligned along the molecular long axis (*X*-axis), suggesting a preferential horizontal orientation on the substrate surface during the deposition process, which is beneficial for improving light outcoupling and device efficiency.^46,79^ Moreover, both emitters feature a central diphenylamine core that is not constrained to adopt a twisted conformation, thereby enhancing the spin–orbit coupling (SOC).^46^ The spin–orbit coupling matrix elements (SOC = 〈S_*n*_|*Ĥ*_SOC_|T_*n*_〉) were calculated as depicted in Fig. S30 and S31. Since S_1_ and T_1_ possess highly similar orbital configurations, their SOC interactions are intrinsically weak. In contrast, T_2_ exhibits a distinctly different electronic configuration, implying that thermally accessible higher-lying triplet states (T_*n*_) may participate as intermediates in the RISC process.^80^ Boltzmann analysis at 298 K indicates that T_2_ contributes approximately 40% of the total triplet population relative to T_1_ for 4F-BOB4, respectively, underscoring its key role in the spin-flip pathway. Moreover, the substantial SOC values between S_1_ and T_2_ (0.93 cm^−1^ for 4F-BOB4) further confirm T_2_-assisted RISC, thereby facilitating efficient exciton up-conversion.

**Fig. 4:**
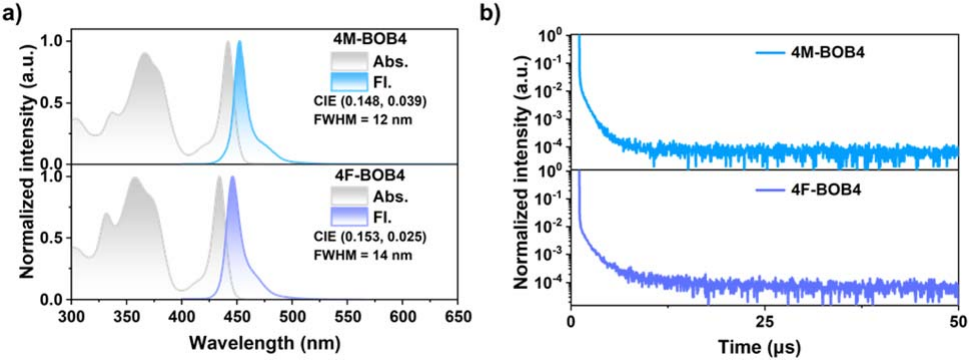
Photophysical properties of 4M-BOB4 and 4F-BOB4. (a) Normalized UV-vis absorption and fluorescence spectra in toluene solution (1 × 10^−5^ M, 298 K); (b) TRPL decay curves of emitters in the 5 wt%-doped DOBNA-OAr film.

The photophysical properties of MR-TADF emitters were investigated in dilute toluene (10^−5^ M) (Fig. 4a), and the key parameters are summarized in Table 1. The UV-vis absorption spectra exhibit distinct absorption bands in the 300–400 nm region, attributable to the n–π*/π–π* transition, and sharp absorption bands in the lowest-energy region (442 and 434 nm) mainly resulted from HOMO to LUMO transitions (Table S5). 4M-BOB4 and 4F-BOB4 displayed deep-blue and violet-blue narrowband emission, peaking at 452 and 442 nm, with remarkably small FWHM values of 12 and 14 nm, corresponding to CIE chromaticity coordinates of (0.148, 0.039) and (0.153, 0.025), respectively. These emitters exhibit small Stokes shifts and pronounced mirror symmetry between their absorption and emission spectra, indicative of minimal conformational reorganization between the S_0_ and S_1_ states. This observation aligns well with their rigid π-conjugated backbones and low reorganization energies, confirming that structural relaxation upon excitation is effectively suppressed. The fluorescence and phosphorescence spectra recorded at 77 K (Fig. S41) reveal an extremely small Δ*E*_ST_ of 0.004 and 0.005 eV for ladder-type emitters 4M-BOB4 and 4F-BOB4, respectively, which are significantly lower than that of the helical structure *f*-DOABNA (0.08 eV). Such minimal Δ*E*_ST_ values are highly favorable for an efficient RISC process. Moreover, nearly identical absorption profiles and only slight red shifts in the emission spectra were observed with increasing solvent polarity (Fig. S42 and S43), indicating that the electronic transitions of these compounds are weakly affected by environmental polarity, underscoring the highly localized nature of the excited states.

**Table 1:** Summary of photophysical properties of emitters

Emitters	*λ*_abs_ a [nm]	*λ*_em_ b [nm]	FWHM^b^ [nm eV^−1^]	Δ*E*_ST_^c^ [eV]	PLQY^d^ [%]	*k*_r_ e [10^7^ s^−1^]	*k*_ISC_ e [10^8^ s^−1^]	*k*_RISC_ e [10^6^ s^−1^]
4M-BOB4	442	452	12/0.08	0.004	85	2.98	1.39	2.22
4F-BOB4	434	442	14/0.09	0.005	82	1.85	1.56	1.59

a Peak wavelength of the absorption maximum in toluene solution (1 × 10^−5^ M, 298 K).

b PL emission spectrum maximum in toluene solution (1 × 10^−5^ M, 298 K).

c Calculated from peaks of the fluorescence and phosphorescence spectra in toluene solution (1 × 10^−5^ M, 77 K) and calculated from Δ*E*_ST_ = S_1_ − T_1_.

d Absolute photoluminescence quantum yield in 5 wt%-doped films of DOBNA-Oar.

e Measured in 5 wt%-doped films of of 4M-BOB4 and 4F-BOB4 in DOBNA-OAr.

To further investigate the solid-state photophysical properties of the emitter, the time-resolved photoluminescence (TRPL) measurements were performed on 4F-BOB4 in doped films (5 wt% in 7-((2′-methyl-[1,1′-biphenyl]-3-yl) oxy)-3,11-di-*o*-tolyl-5,9-dioxa-13*b*-boranaphtho[3,2,1-*de*]anthracene, DOBNA-OAr^81^), as depicted in Fig. 4b and S44. We also measured the PL spectra of films with doping ratios of 2, 5, and 8 wt% in DOBNA-OAr, as provided in Fig. S45 and Table S6. Compared with the toluene solution, 4F-BOB4 doped in the host matrix exhibits negligible changes in the emission peak position, while retaining an ultra-narrow emission band, indicating that its emission properties are minimally affected by variations in the polar environment. The transient PL decay profiles of 5 wt% doped films exhibited a biexponential decay at room temperature, comprising a prompt fluorescence (*τ*_PF_) component with a nanosecond-scale lifetime and a delayed fluorescence (*τ*_DF_) component with a microsecond-scale lifetime, confirming the presence of TADF characteristics. Specifically, the *τ*_PF_ for 4F-BOB4 was determined to be 5.53 ns, while the *τ*_DF_ was 4.85 µs, respectively. Under a nitrogen atmosphere, the doped film of 4F-BOB4 exhibited a high photoluminescence quantum yield (PLQY) of 82%, respectively. Based on the measured PLQY and TRPL, key photophysical parameters were determined using the established methodology^82^ (Table S7). The radiative decay rate constant (*k*_r_) was calculated to be 1.85 × 10^7^ s^−1^ for 4F-BOB4. The intersystem crossing rate (*k*_ISC_) was 1.56 × 10^8^ s^−1^, and the reverse intersystem crossing rate (*k*_RISC_) was 1.59 × 10^6^ s^−1^, respectively. Notably, the relatively high *k*_RISC_ value effectively mitigates efficiency roll-off in the corresponding devices.

**Fig. 5:**
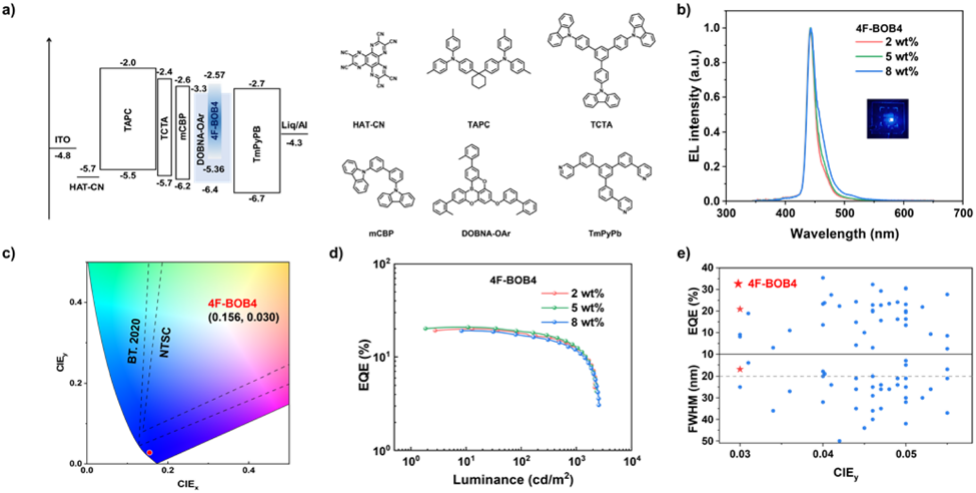
OLED characteristics of 4F-BOB4. (a) Device architecture and the corresponding energy level diagram of the functional materials; (b) EL spectra; (c) CIE coordinates of the devices (NTSC color gamut and BT.2020 color gamut); (d) EQE *versus* luminance characteristic curves; (e) summary plot of the maximum EQE *versus* FWHMs and CIE_*y*_ values (≤0.055) of deep-blue OLEDs reported in the literature (blue circles), with the star indicating the results from this work.

To evaluate the electroluminescence (EL) performance of 4F-BOB4, vacuum-deposited OLED devices were fabricated with the following architecture: indium tin oxide (ITO, 50 nm)/1,4,5,8,9,11-hexaazatriphenylene-hexacarbonitrile (HAT-CN, 10 nm)/1,1-bis[4-[*N*,*N*-di(*p*-tolyl)amino]phenyl]cyclohexane (TAPC, 40 nm)/tris(4-carbazolyl-9-ylphenyl)amine (TCTA, 10 nm)/3,3-bis(*N*-carbazolyl)-1,1′-biphenyl (mCBP, 10 nm)/(*x* = 2, 5, 8 wt% 4F-BOB4:DOBNA-OAr, 20 nm)/1,3,5-tri(*m*-pyridin-3-ylphenyl)benzene (TmPyPB, 45 nm)/Liq (2 nm)/Al (80 nm). Here, HAT-CN, TAPC, TCTA, mCBP, and TmPyPB serve as the hole injection, hole transporting, exciton blocking, electron transporting, and electron injection layers, respectively. DOBNA-OAr was employed as the host material due to its compatible HOMO and LUMO levels with adjacent functional layers and sufficiently high triplet energy (*E*_T_1__ = 2.84 eV), which promotes effective exciton confinement and efficient energy transfer.^31^ The device structure and energy level diagram are depicted in Fig. 5a. The orientation of the emitting dipole moment (EDO) critically influences the light outcoupling efficiency of OLEDs. The angle-dependent p-polarization-resolved photoluminescence spectra (Fig. S45) revealed a high horizontal dipole ratio (*Θ*∥) of 93% for 4F-BOB4. This preferential horizontal alignment, arising from their ladder-type molecular geometry, is anticipated to substantially enhance light extraction and thereby improve electroluminescence performance.

The resulting EL performance of the devices is depicted in Fig. S46, and the corresponding detailed parameters are summarized in Table 2. The emitter exhibits narrowband emission with small FWHM values across all doping ratios (2, 5, and 8 wt%), (Fig. 5b). The EL peaks of 4F-BOB4 remain nearly invariant across doping ratios, closely aligning with their corresponding PL peaks in solution. This exceptional spectral stability stems from the highly sterically hindered architectures, where multiple *tert*-butyl groups along with bulky dimethylbenzene or difluorobenzene moieties suppress π–π interactions and mitigate molecular aggregation. Notably, the 4F-BOB4 based device affords violet-blue emission at 442 nm with an exceptionally narrow FWHM of 16 nm, a CIE_*y*_ of 0.025, and EQE_max/100/1000_ values of 19.1%, 17.9%, and 12.5%, at 2 wt% doping. At an optimal doping of 5 wt%, the device delivers an EQE_max_ of 20.9%, a maximum current efficiency (CE_max_) of 6.8 cd A^−1^, a maximum power efficiency (PE_max_) of 5.9 lm W^−1^, and a low CIE_*y*_ of 0.030, which represents a competitive level among reported violet-blue MR-TADF OLEDs without a sensitizer (CIE_*y*_ ≤ 0.055, Fig. 5e and Table S8). The efficiency roll-off at 1000 cd m^−2^ is 38.8% for 4F-BOB4. Their relatively moderate roll-off behavior in both emitters can be attributed to the relatively rapid RISC rates, which efficiently facilitate the depletion of accumulated triplet excitons and thereby suppress bimolecular quenching pathways, such as triplet–triplet and triplet-polaron annihilation, under high-luminance operating conditions.^83^

**Table 2:** Device performance of the OLEDs based on 4F-BOB4

Emitter	Ratio [wt%]	*λ*_EL_ a [nm]	FWHM^a^ [nm]	CE_max_^b^ [cd A^−1^]	PE_max_^c^ [lm W^−1^]	EQE_max/100/1000_^d^ [%]	Roll-off^e^ [%]	CIE^f^ (*x*, *y*)
4F-BOB4	2	442	16	5.5	4.4	19.1/17.9/12.5	6.3/34.6	0.156, 0.025
	5	443	17	6.8	5.9	20.9/18.8/12.8	10.0/38.8	0.156, 0.030
	8	444	23	8.6	7.3	19.2/17.0/11.7	11.5/39.1	0.154, 0.043

a Maximum electroluminescence wavelength and full width at half maximum of electroluminescence (collected at the same bias of 6 V).

b Maximum current efficiency.

c Maximum power efficiency.

d External quantum efficiency values at maximum, 100 cd m^−2^, and 1000 cd m^−2^.

e External quantum efficiency roll-offs at 100 and 1000 cd m^−2^.

f The CIE coordinates are measured at 6 V.

## Conclusions

In summary, we have developed a molecular design strategy for violet-blue narrowband MR-TADF emitters, which integrate oxygen-bridged triarylboron π-extension with precise heteroatom incorporation to achieve controlled ladder-type fusion. Following this approach, the tetraboron-based emitters, 4M-BOB4 and 4F-BOB4, were successfully synthesized. Their rigid π-conjugated skeletons incorporate strategically embedded boron and oxygen atoms, which locally modulate electron delocalization and finely tune the electronic structure to enable deep-blue narrowband emission. The incorporation of electron-withdrawing fluorine atoms in 4F-BOB4 further induces a hypsochromic shift, leading to improved color purity. 4F-BOB4 exhibits ultra-narrowband emission in toluene solution with a FWHM value of 14 nm. Corresponding OLED devices show violet-blue electroluminescence with narrow FWHMs of 17 nm, along with EQE_max_ values of 20.9%, respectively. Notably, the 4F-BOB4-based device realizes an EQE exceeding 20% at a low CIE_*y*_ coordinate of 0.030, ranking among the highest performances reported for violet-blue MR-TADF emitters. This work demonstrates an effective molecular design concept for constructing blue ultra-narrowband MR-TADF materials and provides a valuable framework for the development of high-performance and wide color gamut OLED emitters.

## Author contributions

Z.-Q. Jiang conceived and supervised the project. J.-R. Wu synthesized and characterized the materials, performed photophysical measurements, and wrote the manuscript. Y.-J. Yang fabricated the OLEDs and characterized the performance of the devices. H.-T. Yuan provided device data assistance. S.-J. Ge performed theoretical calculations. Y.-K. Qu, D.-Y. Zhou, and L.-S. Liao participated in discussions. H.-X. Jiang and Y. Liu provided data support. All authors engaged in result analysis and provided feedback on the manuscript.

## Conflicts of interest

The authors declare no conflict of interest.

## Supplementary Material

SC-017-D5SC09014H-s001

SC-017-D5SC09014H-s002

## Data Availability

CCDC 2430120, 2430121 and 2452651 contain the supplementary crystallographic data for this paper.^84*a*–*c*^ The data that support the findings of this study are available within the main text and its supplementary information (SI). Supplementary information is available. See DOI: https://doi.org/10.1039/d5sc09014h.
