# Supplementary material for: Ladder-type π-conjugated frameworks with multi-heteroatom modulation for narrowband violet-blue multiple-resonance emitters with a low CIEy of 0.03
Source: Chem Sci. 2026 Jan 26;17(11):5638–47. doi: 10.1039/d5sc09014h (PMC12834219; doi:10.1039/d5sc09014h)
Supplement: SC-017-D5SC09014H-s001 [file SC-017-D5SC09014H-s001.pdf]

Supplementary Information (SI) for Chemical Science.
This journal is © The Royal Society of Chemistry 2026

Supporting Information

Ladder-Type π-Conjugated Frameworks with Multi-heteroatom Modulation for Narrowband Violet-Blue Multiple-Resonance Emitters with Low CIE_y of 0.03

Jian-Rong Wu[1,#] *Yue-Jian Yang*[1,#] *Hai-Tian Yuan*[1], *Shi-Jie Ge*[1], *Yang-Kun Qu*[1], *Hai-Xiao Jiang*[1], *Yin Liu*[1], *Dong-Ying Zhou*[1], *Liang-Sheng Liao*[1,2], *Zuo-Quan Jiang*[1*]

[1] State Key Laboratory of Bioinspired Interfacial Materials Science,
Institute of Functional Nano & Soft Materials (FUNSOM),
Soochow University, Suzhou, 215123, Jiangsu, PR China
E-mail: zqjiang@suda.edu.cn
[2] Macao Institute of Materials Science and Engineering, Macau University of Science and Technology, Taipa, Macau 999078, China

[#]These authors contributed equally to this work.

Contents

1. General Information S2

2. Experimental Section S3

3. Single Crystal Data S17

4.Thermal and Electrochemical Characterization S22

5. Computational Methods S23

6. Photophysical properties S30

7. Device Fabrication and Measurement S33

8. Reference S36

1. General Information

All reagents and solvents were commercially available and were used without further purification unless otherwise noted. NMR spectra were taken on Bruker AVANCE III HD (400 MHz). Chemical shifts are reported in parts per million (ppm) relative to traces of $CHCl_3$ in the corresponding deuterated solvents. Matrix-assisted laser desorption/ionization time-of-flight mass spectrometry (MALDI-TOF-MS) was performed on Bruker Autoflex II/Compass 1.0. Thermogravimetric analysis (TGA) was performed on a TA SDT 2960 from 25 °C to 800 °C under dry nitrogen at a heating rate of 10 °C min^{-1}. UV-vis absorption pectra were recorded on a Shimadzu UV 2600 spectrophotometer. Photoluminescence (PL) spectra and phosphorescent spectra were performed on Hitachi F-4600 fluorescence spectrophotometer. The absolute PLQY values were measured using Hamamatsu C9920-02G in an integrating sphere under a nitrogen atmosphere. Cyclic voltammagrams (CV) were obtained using a glassy carbon working electrode, a platinum counter electrode, and an Ag/AgCl reference electrode tested on CHI660E station. The solvent in the measurement was CH_2Cl_2, and the supporting electrolyte was 0.1 M tetrabutylammonium hexafluorophosphate. Ferrocene was added as a calibrant after each set of measurements, and all potentials reported were quoted regarding the ferrocene-ferrocenium (Fc/Fc^+) couple at a scan rate of 100 mV/s.

2. Experimental Section

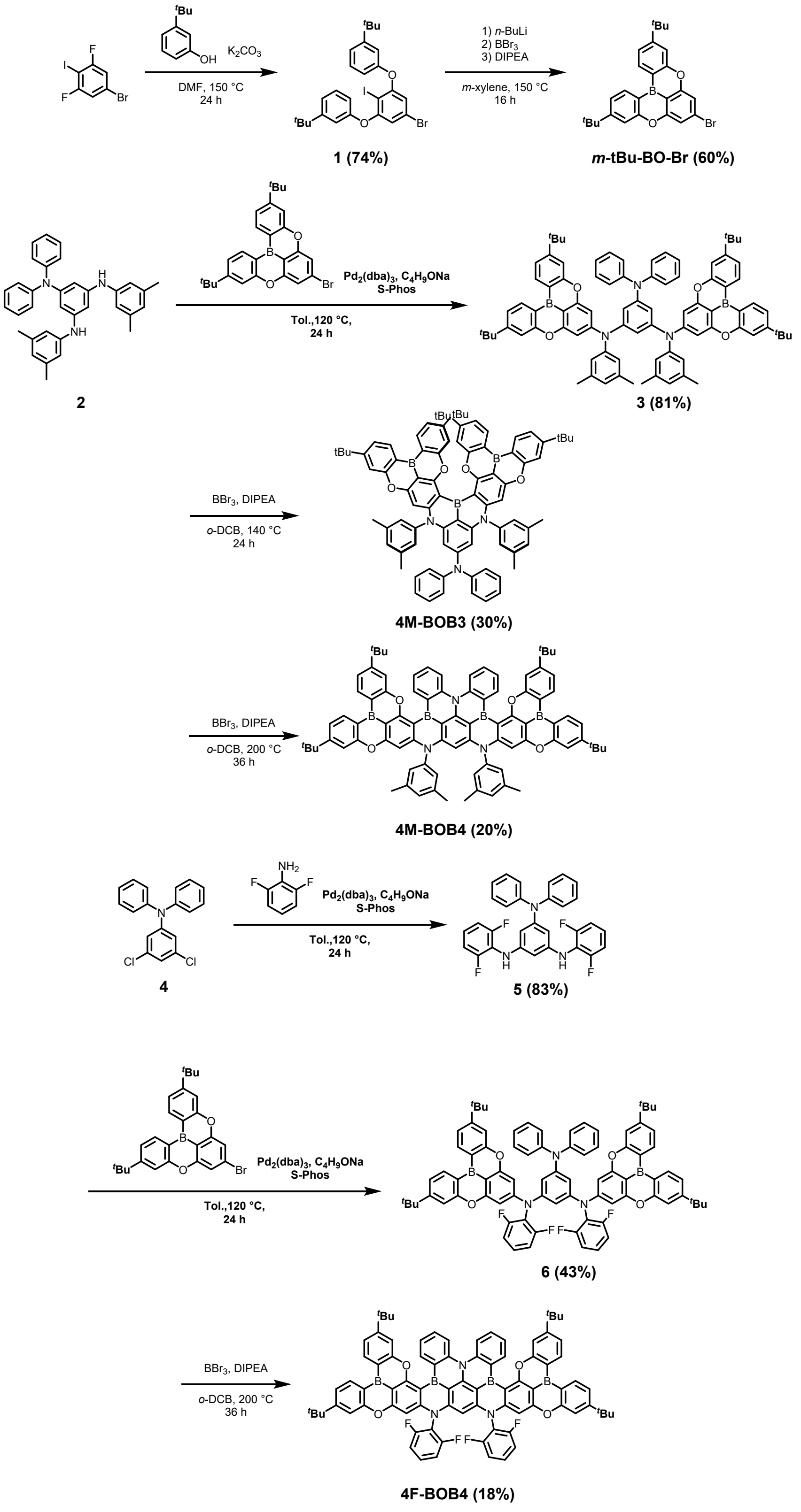

Scheme S1. Synthetic procedures of **4M-BOB3**, **4M-BOB4** and **4F-BOB4**.

The vital precursor **2** and **4** were synthesized via Buchwald-Hartwig coupling reaction, which procured from readily accessible sources as detailed in prior literature. [S1]

Synthesis of **3,3'-((5-bromo-2-iodo-1,3-phenylene)bis(oxy))bis(*tert*-butylbenzene) (1)**

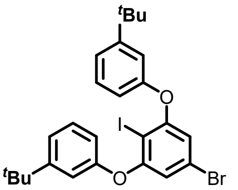

A dry 250 mL glass vial was charged with 5-bromo-1,3-difluoro-2-iodobenzene (10.00 g, 31.36 mmol), 3-(*tert*-butyl) phenol (11.78 g, 78.40 mmol), potassium carbonate (17.34 g, 125.44 mmol). Anhydrous DMF (100 mL) was added to the vial and the mixture was bubbled with argon for 3 minutes. The vial was quickly sealed and heated at 150 °C for 24 hours. After cooling down to room temperature, the reaction mixture was diluted with dichloromethane (200 mL) and washed with water (200 mL) and dired over Na_2SO_4. The solvent was removed by rotatory evaporation and the crude product was purified by silicagel column chromatography (petroleum ether) to give the product as white solid (13.50 g, 74%). ^{1}H NMR (400 MHz, $CDCl_3$) δ (ppm) = 7.36 (t, J = 7.9 Hz, 2H), 7.30-7.24 (m, 2H), 7.17 (d, J = 2.1 Hz, 2H), 6.86 (ddd, J = 8.0, 2.5, 1.1 Hz, 2H), 6.66 (s, 2H), 1.37 (s, 18H). ^{13}C NMR (100 MHz, $CDCl_3$) δ (ppm) = 159.66, 155.67, 154.12, 129.65, 122.97, 121.87, 117.25, 116.50, 115.21, 81.01, 35.04, 31.43. HRMS (ESI) *m/z* calcd. $[M]^+$ for $C_{26}H_{28}BrIO_2$: 578.0317, found: 578.0509.

Synthesis of **7-bromo-3,11-di-tert-butyl-5,9-dioxa-13b-boranaphtho[3,2,1-*de*]anthracene (*m*-tBu-BO-Br)**

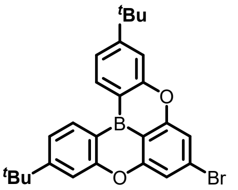

A solution of butyllithium in hexane (1.6 M, 12.94 mL, 20.71 mmol) was added slowly to a solution of intermediate **1** (10.00 g, 17.26 mmol) in *m*-xylene (150 mL) at -40 ºC under argon atmosphere and maintained for 2 hours, boron tribromide (2.50 mL, 25.89 mmol) was added at -40 ºC. After the reaction mixture was allowed to warm to room temperature and stirred for 1 h. *N*,*N*-diisopropylethylamine (6.19 mL, 34.52 mmol) was added at 0 ºC, the reaction mixture was stirred at 150 ºC for 16 h. After cooling down to room temperature, adding ethanol to quench the reaction. The solvent was removed by rotatory evaporation the reaction mixture was diluted with dichloromethane (200 mL) and washed with water (200 mL) and dired over Na_2SO_4. and the crude product was purified by silicagel column chromatography (petroleum ether) to give the product as white solid (4.80 g, 60%). ^{1}H NMR (400 MHz, $CDCl_3$) δ (ppm) = 8.58 (dd, J = 8.1, 1.7 Hz, 2H), 7.52 (d, J = 1.8 Hz, 2H), 7.45 (dd, J = 8.1, 1.9 Hz, 2H), 7.35 (d, J = 1.1 Hz, 2H), 1.44 (s, 18H). ^{13}C NMR (100 MHz, $CDCl_3$) δ (ppm) = 160.60, 158.14, 157.96,

134.33, 128.00, 121.11, 115.05, 112.10, 35.44, 31.24. HRMS (ESI) *m/z* calcd. $[M]^+$ for $C_{26}H_{26}BBrO_2$: 460.1209, found: 460.1218.

Synthesis of ***N*^{1},*N*^{3}-bis(3,11-di-tert-butyl-5,9-dioxa-13b-boranaphtho[3,2,1-*de*]anthracen-7-yl)-*N*^{1},*N*^{3}-bis(3,5-dimethylphenyl)-*N*^{5},*N*^{5}-diphenylbenzene-1,3,5-triamine (3)**

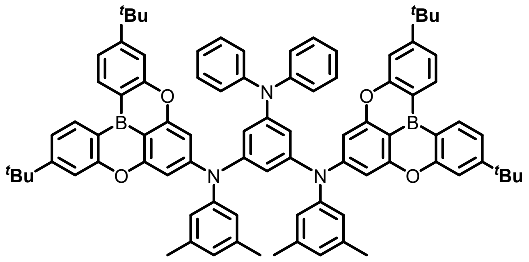

A dry 120 mL glass vial was charged with intermediate **2** (1.00 g, 2.07 mmol), intermediate ***m*-tBu-BO-Br** (2.38 g, 5.17 mmol), sodium *tert*-butoxide (811 mg, 8.27 mmol), tris(dibenzylideneacetone)dipalladium (94 mg, 0.10 mmol), S-Phos (127 mg, 0.31 mmol). Anhydrous toluene (20 mL) to was added to the vial and the mixture was bubbled with argon for 3 minutes. The vial was quickly sealed and heated at 120°C for 24 hours. After cooling down to room temperature, the reaction mixture was diluted with dichloromethane (200 mL) and washed with water (200 mL) and dried over Na_2SO_4. The solvent was removed by rotatory evaporation and the crude product was purified by silicagel column chromatography (dichloromethane/petroleum ether = 1:6) to give the product as faint yellow solid (2.10 g, 81%). ^{1}H NMR (400 MHz, $CDCl_3$) δ (ppm) = 8.54(d, *J* = 8.1 Hz, 4H), 7.47 (d, *J* = 1.9 Hz, 4H), 7.40 (dd, *J* = 8.1, 1.9 Hz, 4H), 7.18 (h, *J* = 6.5 Hz, 8H), 6.88-6.83 (m, 6H), 6.77 (s, 2H), 6.74 (d, *J* = 1.8 Hz, 2H), 6.72 (s, 4H), 6.63 (t, *J* = 1.9 Hz, 1H), 2.28 (s, 12H), 1.43 (s, 36H). ^{13}C NMR (100 MHz, $CDCl_3$) δ (ppm) = 160.88, 158.56, 157.00, 153.78, 149.30, 148.30, 147.15, 146.00, 139.33, 134.15, 129.30, 127.08, 124.58 (d, J = 3.5 Hz), 123.24, 120.39, 120.08, 117.13, 116.41, 114.72, 109.75, 100.29, 35.31, 31.30, 21.47. MS (MALDI-TOF) *m/z* $[M]^+$ calcd. for $C_{86}H_{83}B_2N_3O_4$: 1243.65, found: 1243.32.

Synthesis of **4M-BOB3**

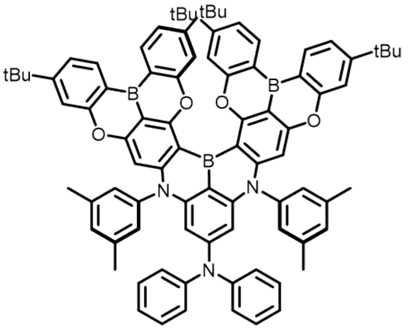

A dry 120 mL glass vial was charged with intermediate **3** (100 mg, 0.08 mmol), anhydrous o-dichlorobenzene (10 mL) and boron tribromide (0.77 mL, 0.80 mmol) under the protection of argon stirred in an oil-bath at 140 °C for 24 hours. As the reaction cooled to 0 ºC, N, N-diisopropylethylamine (0.14 mL, 0.80 mmol) was added

to the mixture. Then the mixture was diluted with dichloromethane (100 mL), washed with water (100 mL) and dried over Na_2SO_4. The solvent was removed by rotatory evaporation and the crude product was purified by silica gel column chromatography (dichloromethane/petroleum ether = 1:4) to give the product **4M-BOB3** as yellow solid (31 mg, 30%). ^{1}H NMR (400 MHz, $CDCl_3$) δ (ppm) =8.49 (d, J = 8.1 Hz, 2H), 8.32 (d, J = 8.1 Hz, 2H), 7.44 (d, J = 1.8 Hz, 2H), 7.35 (dd, J = 8.1, 1.9 Hz, 2H), 7.15 (t, J = 7.7 Hz, 4H), 7.08-6.92 (m, 14H), 6.58 (s, 2H), 6.36 (d, J = 1.8 Hz, 2H), 5.91 (s, 2H), 2.35 (s, 12H), 1.55 (s, 6H), 1.42 (s, 18H), 0.85 (s, 18H). ^{13}C NMR (100 MHz, $CDCl_3$) δ (ppm) = 163.76, 160.94, 160.60, 159.62, 156.58, 156.02, 153.28, 150.69, 147.06, 146.68, 141.74, 134.27, 133.17, 130.15, 128.86, 125.43, 123.38, 120.17, 119.96, 114.45, 101.01, 96.09, 35.28, 34.67, 31.33, 30.72, 21.48. MS (MALDI-TOF) *m/z* $[M]^+$ calcd. for $C_{86}H_{80}B_3N_3O_4$: 1251.64, found: 1251.48.

Synthesis of **4M-BOB4**

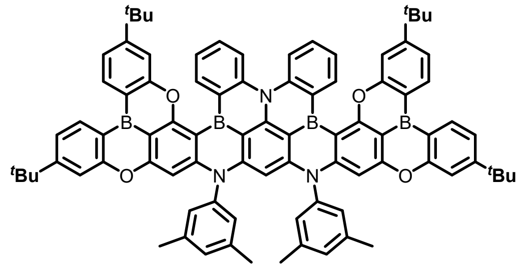

A dry 120 mL glass vial was charged with intermediate **3** (1.00 g, 0.80 mmol), anhydrous o-dichlorobenzene (20 mL) and boron tribromide (1.55 mL, 16.07 mmol) under the protection of argon stirred in an oil-bath at 200 °C for 36 hours. As the reaction cooled to 0 ºC, N, N-diisopropylethylamine (2.89 mL, 16.07 mmol) was added to the mixture. Then the mixture was diluted with dichloromethane (200 mL), washed with water (200 mL) and dried over Na_2SO_4. The solvent was removed by rotatory evaporation and the crude product was purified by silica gel column chromatography (dichloromethane/petroleum ether = 1:4) to give the product **4M-BOB4** as yellow solid (210 mg, 20%). ^{1}H NMR (400 MHz, $CDCl_3$) δ (ppm) = 8.98 (dd, J = 7.8, 1.7 Hz, 2H), 8.70 (d, J = 8.1 Hz, 2H), 8.63 (d, J = 8.0 Hz, 2H), 8.50 (d, J = 8.5 Hz, 2H), 7.79 (d, J = 1.8 Hz, 2H), 7.64 (ddd, J = 8.6, 7.0, 1.7 Hz, 2H), 7.54 (dd, J = 8.1, 1.9 Hz, 2H), 7.46-7.36 (m, 6H), 7.17 (s, 2H), 6.85 (s, 4H), 6.65 (s, 2H), 5.46 (s, 2H), 2.41 (s, 6H), 2.36 (s, 6H), 1.52 (s, 18H), 1.42 (s, 18H). ^{13}C NMR (100 MHz, $CDCl_3$) δ (ppm) = 163.03, 160.99, 160.70, 159.74, 157.28, 157.25, 152.80, 149.08, 146.83, 145.29, 141.90, 141.02, 140.27, 136.56, 134.41, 134.01, 129.94, 129.39, 127.76, 126.85, 123.80, 121.99, 120.79, 120.56, 115.74, 114.63, 106.96, 97.34, 95.85, 35.54, 35.36, 31.46, 31.30, 21.51, 21.46. MS (MALDI-TOF) *m/z* $[M]^+$ calcd. for $C_{86}H_{77}B_4N_3O_4$: 1259.62, found: 1259.37.

Synthesis of ***N*^{1},*N*^{3}-bis(2,6-difluorophenyl)-*N*^{5},*N*^{5}-diphenylbenzene-1,3,5-triamine (5)**

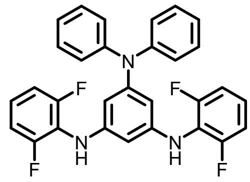

A dry 120 mL glass vial was charged with intermediate **4** (1.50 g, 4.77 mmol), 2,6-difluoroaniline (1.54 g, 11.93 mmol), sodium *tert*-butoxide (1.38 g, 14.32 mmol), tris(dibenzylideneacetone)dipalladium (174 mg, 0.19 mmol), S-Phos (273 mg, 0.57 mmol). Anhydrous toluene (20 mL) was added to the vial and the mixture was bubbled with argon for 3 minutes. The vial was quickly sealed and heated at 120 °C for 24 hours. After cooling down to room temperature, the reaction mixture was diluted with dichloromethane (200 mL) and washed with water (200 mL) and dired over Na_2SO_4. The solvent was removed by rotatory evaporation and the crude product was purified by silicagel column chromatography (dichloromethane/petroleum ether = 1:2) to give the product as white solid (2.00 g, 83%). ^{1}H NMR (400 MHz, DMSO) δ (ppm) = 7.66 (d, J = 2.5 Hz, 2H), 7.29-7.21 (m, 4H), 7.14-6.93 (m, 12H), 5.82 (q, J = 1.9 Hz, 2H), 5.74 (s, 1H). ^{13}C NMR (100 MHz, DMSO) δ (ppm) = 158.62, 158.57, 156.17, 156.11, 148.11, 147.16, 146.27, 129.18, 124.52, 124.42, 124.33, 123.85, 122.53, 118.78, 118.62, 118.47, 112.10, 112.04, 111.92, 111.86, 101.21, 95.36. HRMS (ESI) *m/z* calcd. $[M]^+$ for $C_{30}H_{21}F_4N_3$: 499.1672, found: 499.1649.

Synthesis of ***N*^{1},*N*^{3}-bis(3,11-di-tert-butyl-5,9-dioxa-13b-boranaphtho[3,2,1-*de*]anthracen-7-yl)-*N*^{1},*N*^{3}-bis(2,6-difluorophenyl)-*N*^{5},*N*^{5}-diphenylbenzene-1,3,5-triamine (6)**

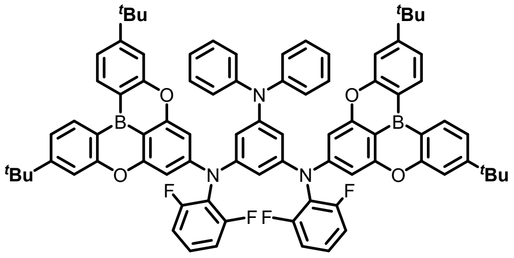

A dry 120 mL glass vial was charged with intermediate **5** (1.00 g, 2.00 mmol), intermediate ***m*-tBu-BO-Br** (2.03 g, 4.40 mmol), sodium *tert*-butoxide (785 mg, 8.03 mmol), tris(dibenzylideneacetone)dipalladium (91 mg, 0.10 mmol), S-Phos (123 mg, 0.30 mmol). Anhydrous toluene (20 mL) to was added to the vial and the mixture was bubbled with argon for 3 minutes. The vial was stirred in an oil bath at 120 °C for 24 hours. After cooling down to room temperature, the reaction mixture was diluted with dichloromethane (150 mL), washed with water (150 mL) and dried over Na_2SO_4. The solvent was removed by rotatory evaporation and the crude product was purified by silica gel column chromatography (dichloromethane/petroleum ether = 1:6) to give the product as white solid (1.10 g, 43%). ^{1}H NMR (400 MHz, $CDCl_3$) δ (ppm) = 8.33 (d, J = 8.1 Hz, 4H), 7.26 (d, J = 1.8 Hz, 4H), 7.19 (dd, J = 8.1, 1.8 Hz, 4H), 7.08-6.94 (m, 10H), 6.78 (t, J = 8.1 Hz, 4H), 6.72-6.67 (m, 2H), 6.66 (d, J = 2.0 Hz, 2H), 6.44 (s, 4H), 6.44-6.42 (m, 1H), 1.22 (s, 36H). ^{13}C NMR (100 MHz, $CDCl_3$) δ (ppm)= 161.65,

160.87, 159.13, 158.77, 157.12, 152.27, 149.49, 147.09, 146.43, 134.19, 129.37, 128.25, 124.64, 123.40, 120.48, 115.38, 114.70, 113.66, 112.98, 112.77, 98.07, 35.31, 31.29. MS (MALDI-TOF) *m/z* $[M]^+$ calcd. for $C_{82}H_{71}B_2F_4N_3O_4$: 1259.55, found: 1259.37.

Synthesis of **4F-BOB4**

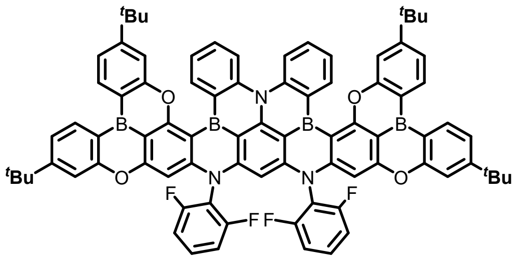

A dry 120 mL glass vial was charged with intermediate **6** (1.00 g, 0.79 mmol), anhydrous *o*-dichlorobenzene (20 mL) and boron tribromide (1.53 mL, 15.87 mmol) under the protection of argon stirred in an oil-bath at 200 °C for 36 hours. As the reaction cooled to 0 ºC, *N*, *N*-diisopropylethylamine (2.85 mL, 15.87 mmol) was added to the mixture. Then the mixture was diluted with dichloromethane (200 mL), washed with water (200 mL) and dried over Na_2SO_4. The solvent was removed by rotatory evaporation and the crude product was purified by silica gel column chromatography (dichloromethane/petroleum ether = 1:4) to give the product **4F-BOB4** as yellow solid (190 mg, 18%) ^{1}H NMR (400 MHz, $CDCl_3$) δ (ppm) = 9.00 (dd, J = 7.8, 1.7 Hz, 2H), 8.70 (d, J = 8.1 Hz, 2H), 8.66-8.61 (m, 2H), 8.50 (d, J = 8.5 Hz, 2H), 7.78 (d, J = 1.8 Hz, 2H), 7.65 (ddd, J = 8.6, 6.9, 1.7 Hz, 2H), 7.58-7.49 (m, 4H), 7.47-7.37 (m, 6H), 7.19 (t, J = 8.4 Hz, 2H), 7.09 (t, J = 8.4 Hz, 2H), 6.63 (s, 2H), 5.55 (s, 1H), 1.52 (s, 18H), 1.42 (s, 18H). ^{13}C NMR (100 MHz, $CDCl_3$) δ (ppm) = 163.21, 161.00, 160.66, 157.50, 157.44, 151.68, 148.42, 146.86, 136.70, 134.47, 134.06, 129.79, 123.74, 120.95, 120.76, 115.75, 114.64, 95.45, 95.14, 35.57, 35.37, 31.45, 31.28. MS (MALDI-TOF) *m/z* $[M]^+$ calcd. for $C_{82}H_{71}B_4F_4N_3O_4$: 1275.52, found: 1275.35.

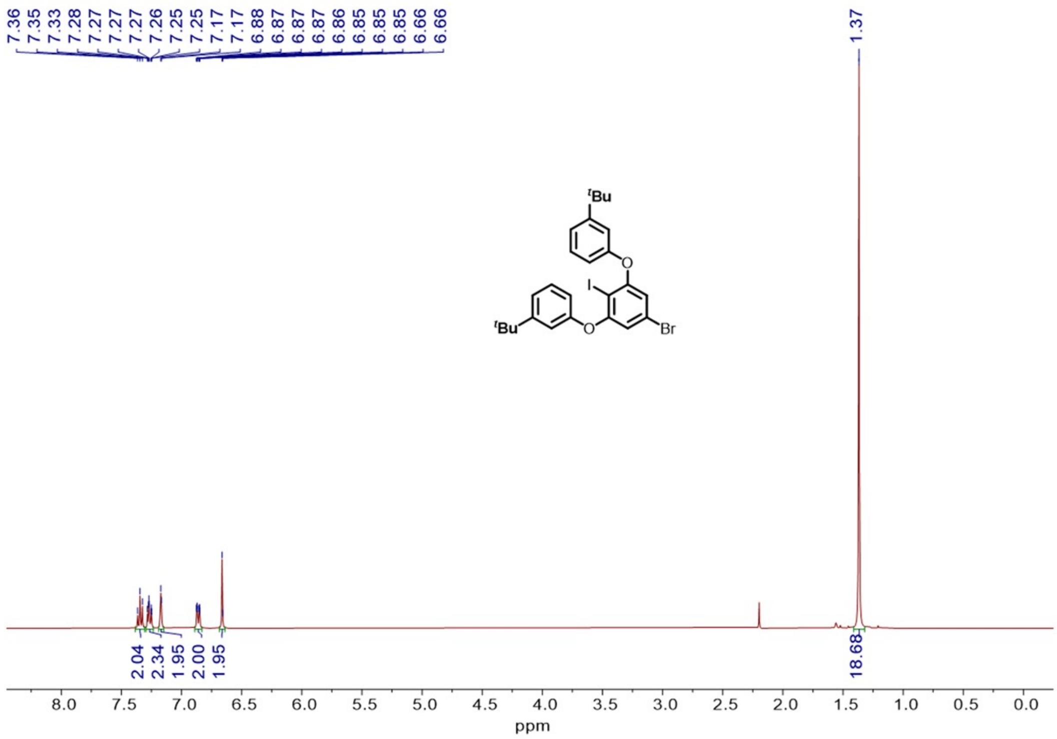

Figure S1. ^{1}H NMR spectrum (400 MHz, $CDCl_3$) of intermediate **1**.

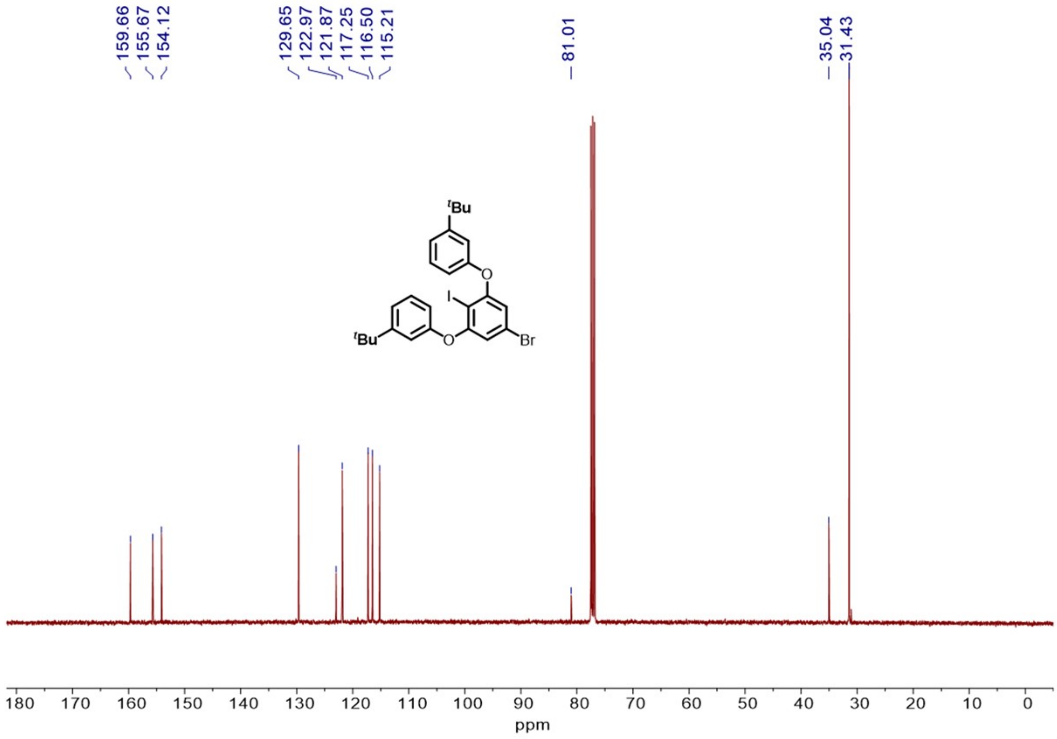

Figure S2. ^{13}C NMR spectrum (100 MHz, $CDCl_3$) of intermediate **1**.

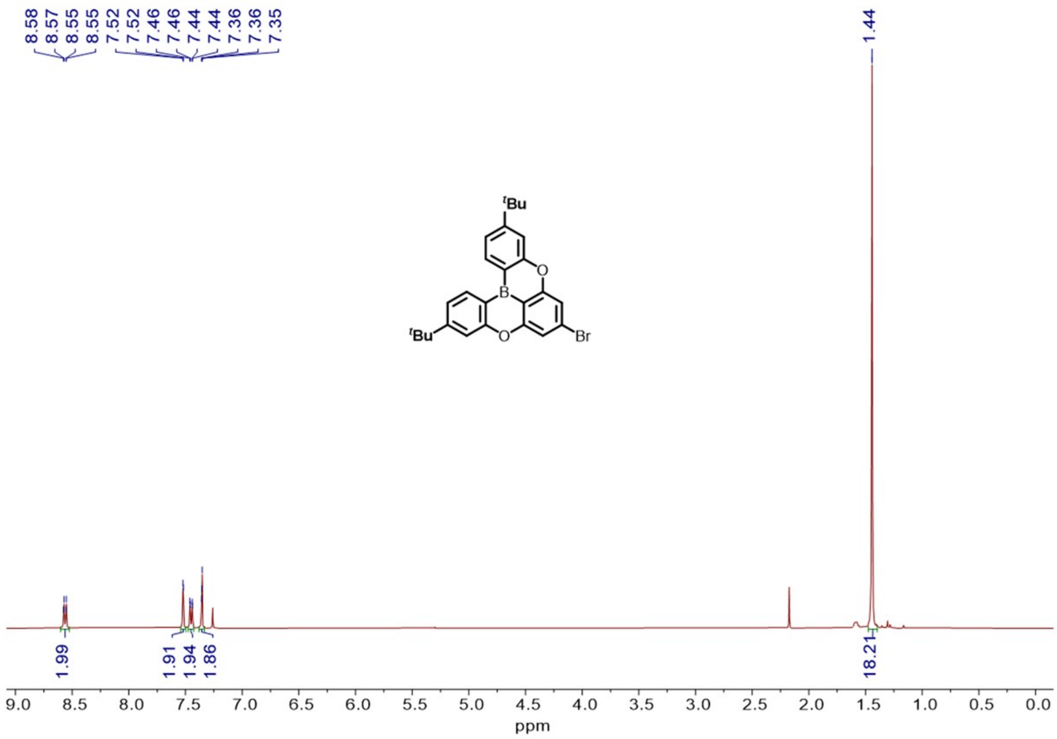

Figure S3. ^{1}H NMR spectrum (400 MHz, $CDCl_3$) of ***m*-tBu-BO-Br**.

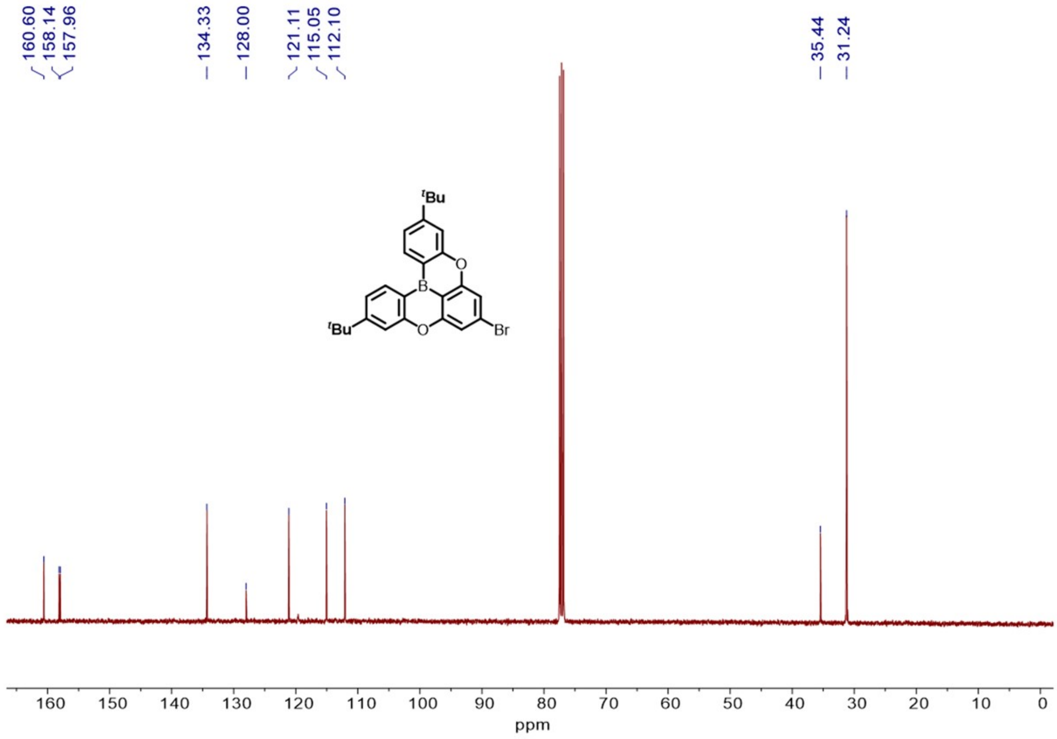

Figure S4. ^{13}C NMR spectrum (100 MHz, $CDCl_3$) of **m-tBu-BO-Br**

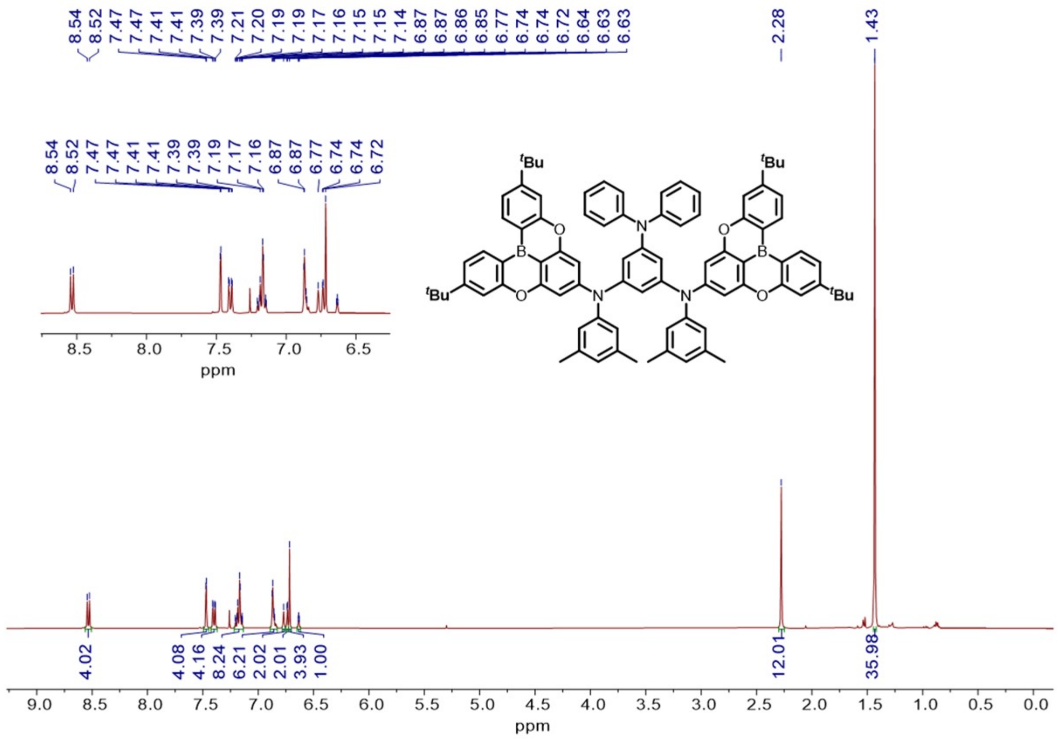

Figure S5. ^{1}H NMR spectrum (400 MHz, $CDCl_3$) of intermediate **3**.

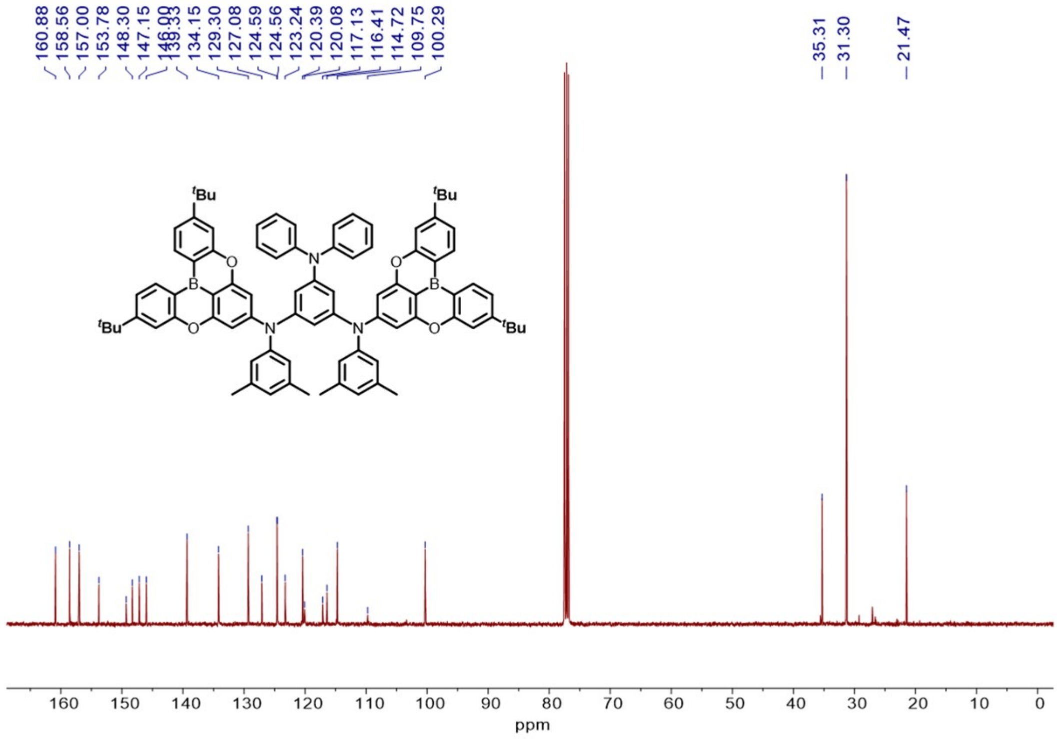

Figure S6. ^{13}C NMR spectrum (100 MHz, $CDCl_3$) of intermediate **3**.

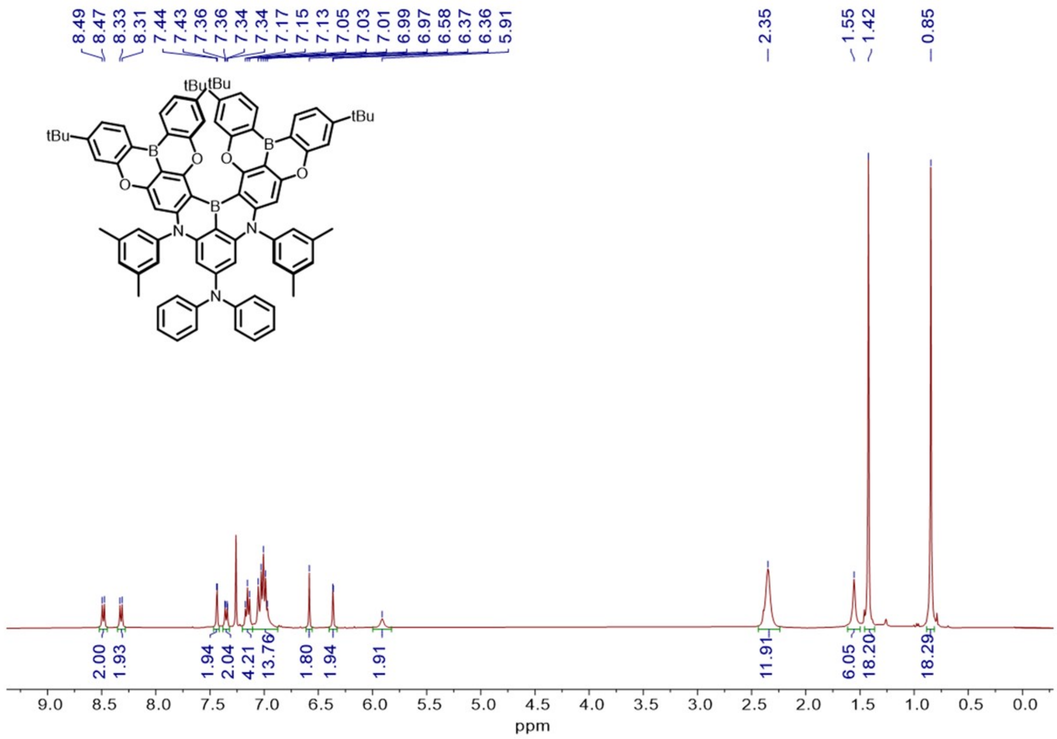

Figure S7. ^{1}H NMR spectrum (400 MHz, $CDCl_3$) of **4M-BOB3**.

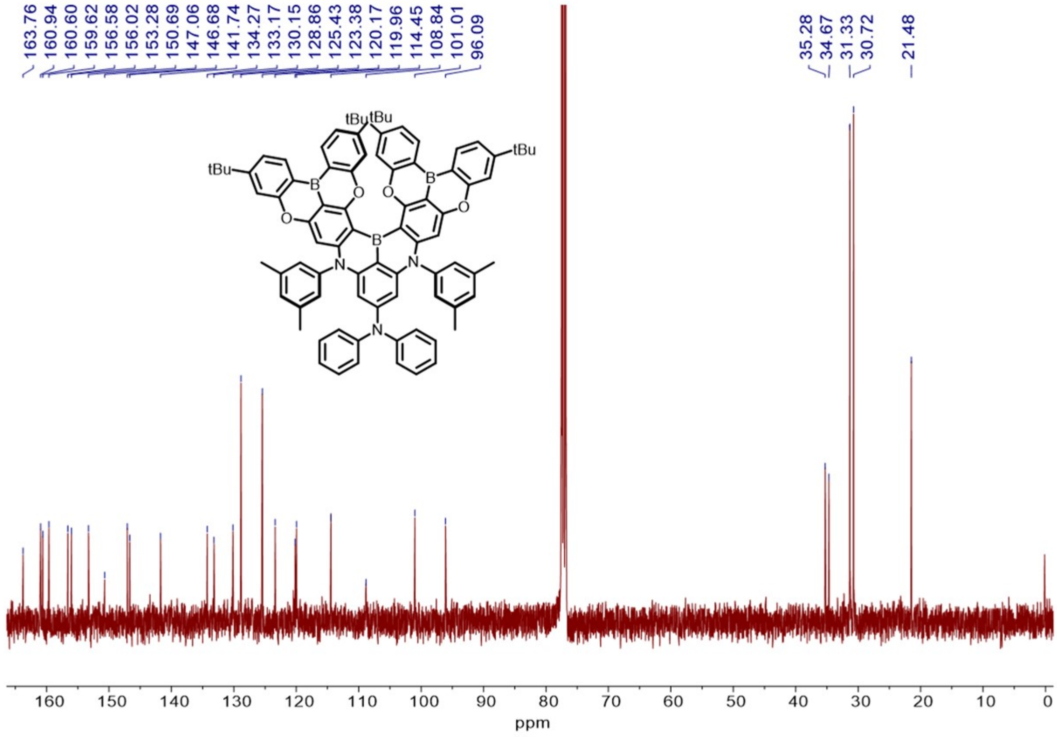

Figure S8. ^{13}C NMR spectrum (100 MHz, $CDCl_3$) of **4M-BOB3**.

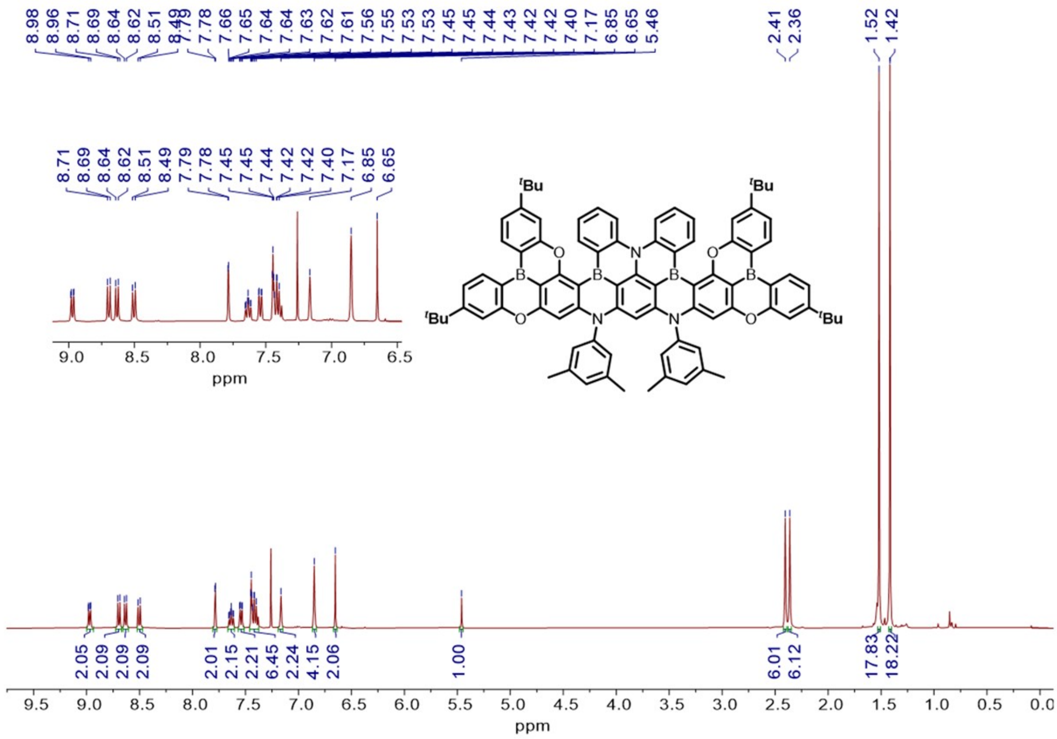

Figure S9. ^{1}H NMR spectrum (400 MHz, $CDCl_3$) of **4M-BOB4**.

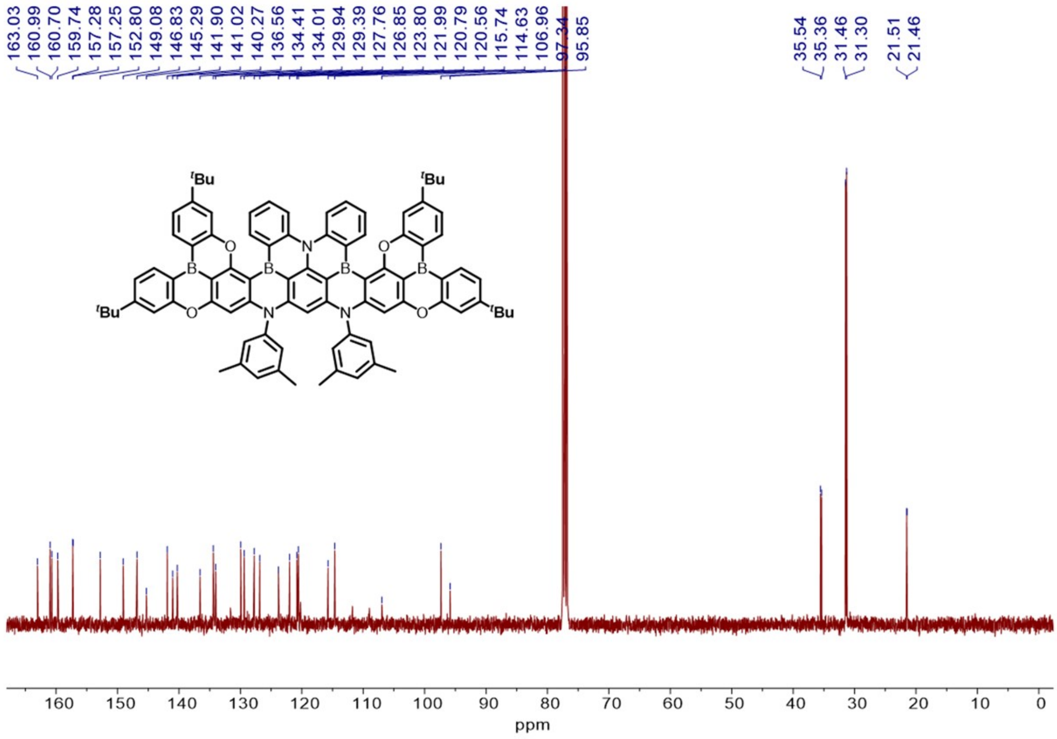

Figure S10. ^{13}C NMR spectrum (100 MHz, $CDCl_3$) of **4M-BOB4**.

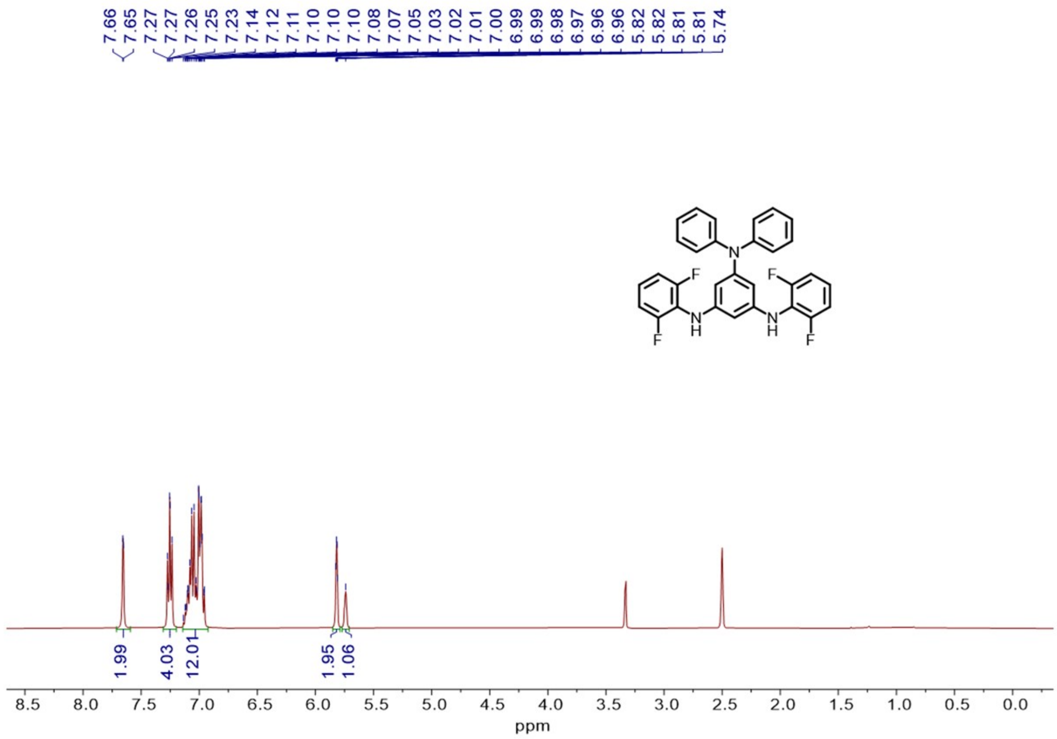

Figure S11. ^{1}H NMR spectrum (400 MHz, DMSO) of intermediate **5**.

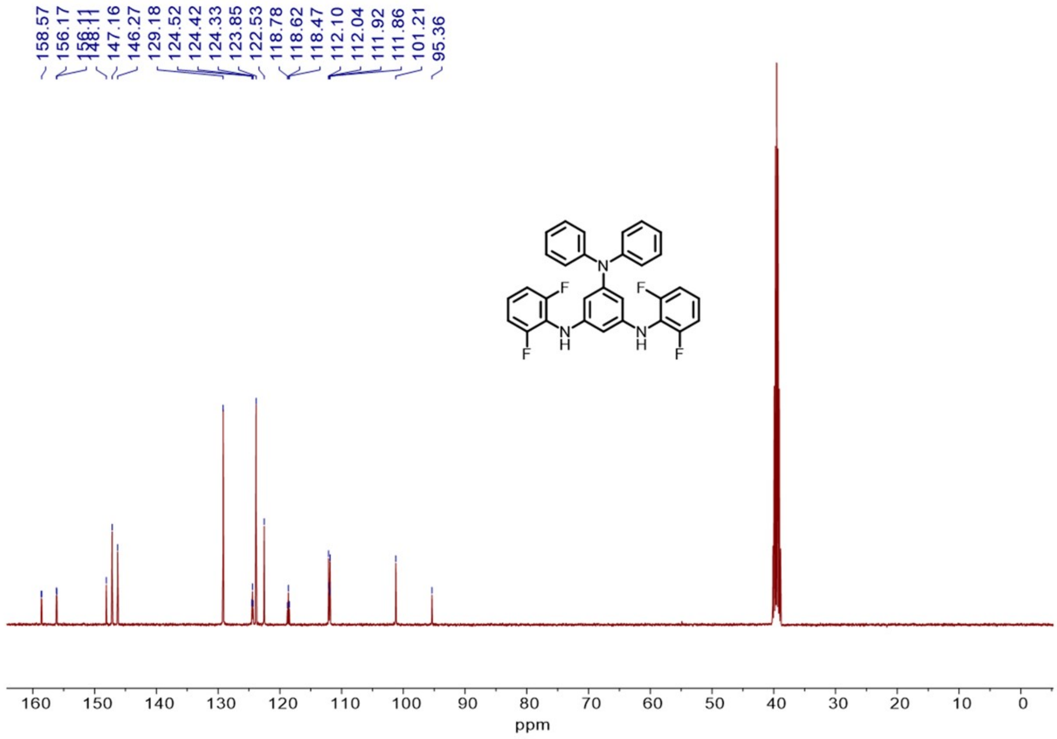

Figure S12. ^{13}C NMR spectrum (100 MHz, DMSO) of intermediate **5**.

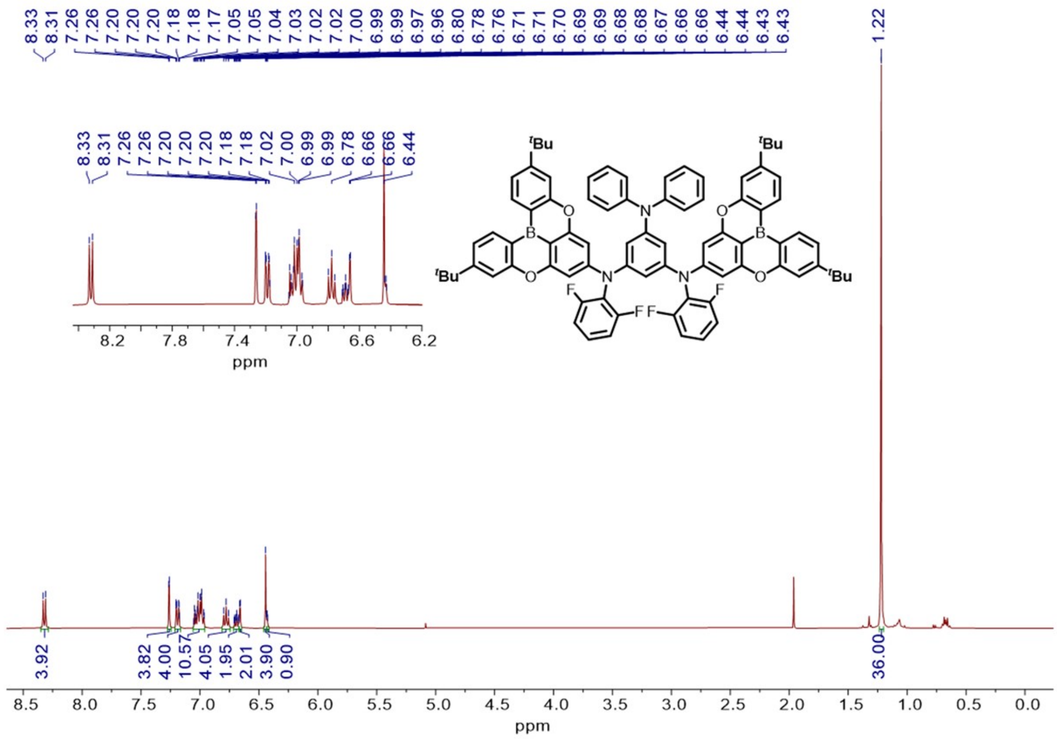

Figure S13. ^{1}H NMR spectrum (400 MHz, $CDCl_3$) of **6**.

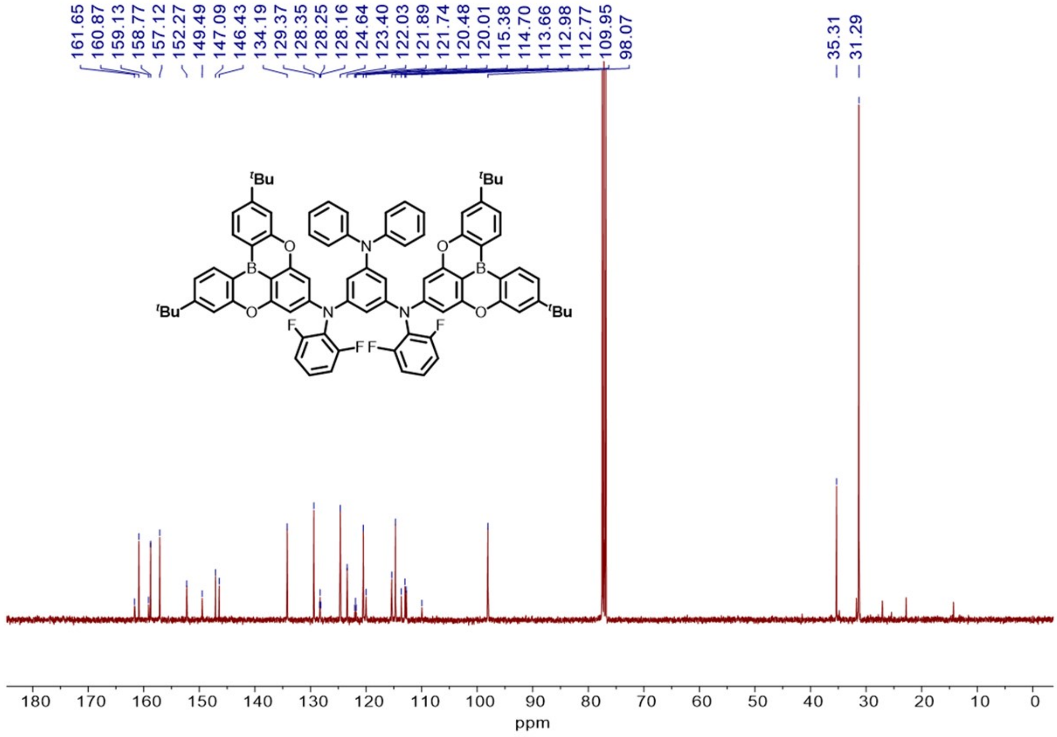

Figure S14. ^{13}C NMR spectrum (100 MHz, $CDCl_3$) of **6**.

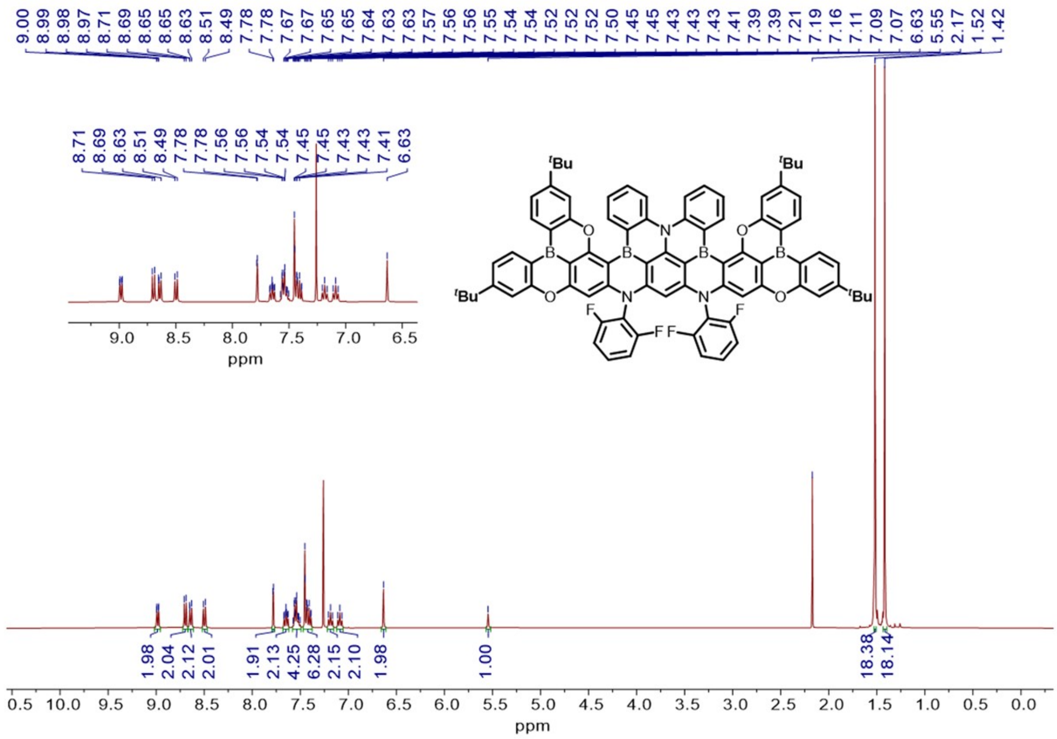

Figure S15. ^{1}H NMR spectrum (400 MHz, $CDCl_3$) of **4F-BOB4**.

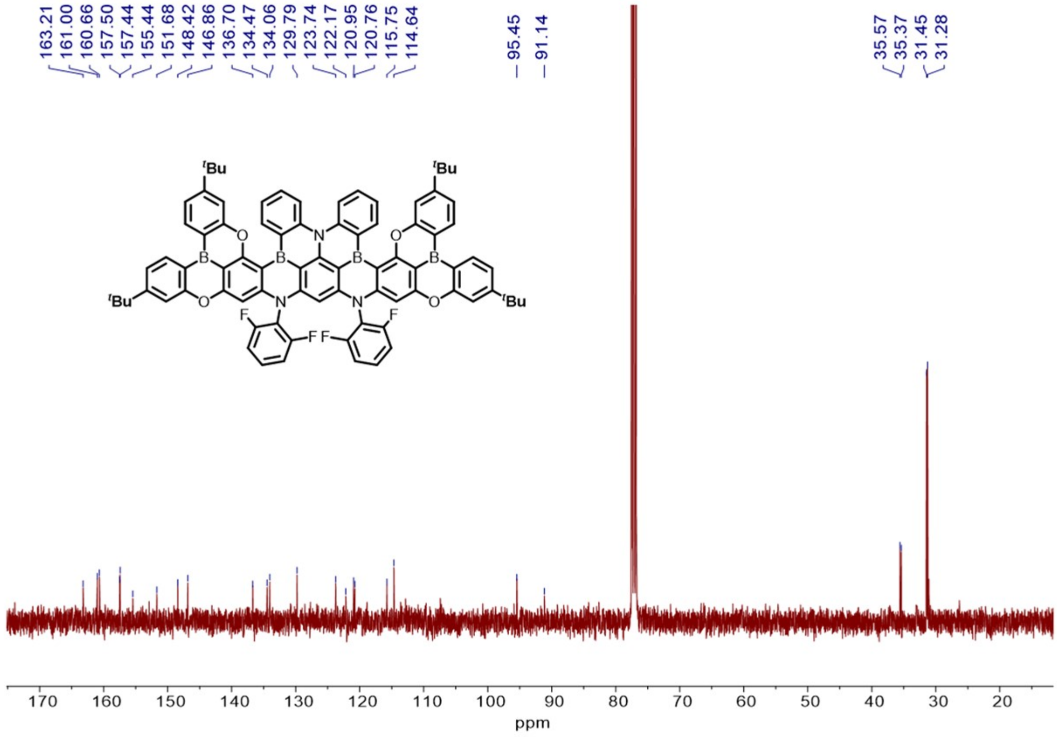

Figure S16. ^{13}C NMR spectrum (100 MHz, $CDCl_3$) of **4F-BOB4**.

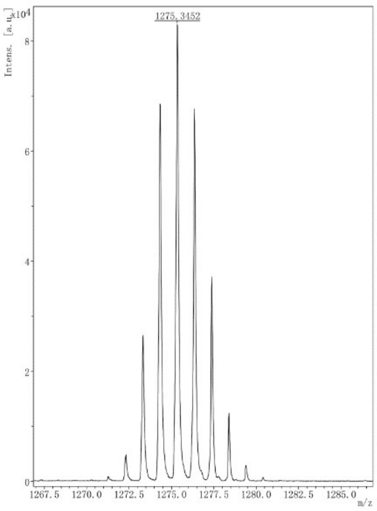

Figure S17. MALDI-TOF spectrum of 4F-BOB4.

3. Single Crystal Data

Crystal data collection was performed on a Bruker D8-Venture diffractometer with a Turbo X-ray Source (Cu-Kα radiation, λ = 1.54178Å) adopting the direct drive rotating anode technique and a CMOS detector at low temperatures. The crystallographic information in CIF format have been deposited at the Cambridge Crystallographic Data Center (CCDC) under deposition number 2452651, 2430120 and 2430121 for **4M-BOB3**, **4M-BOB4** and **4F-BOB4** via www.ccdc.cam.ac.uk/data_request/cif.

Table S1. Crystal data and structure refinement for 4M-BOB4.

Empirical formula	$C_{86}H_{77}B_4N_3O_4$
Formula weight	1259.74
Temperature/K	170
Crystal system	triclinic
Space group	P-1
a/Å	18.8242(8)
b/Å	20.1227(9)
c/Å	22.8761(10)
α/°	92.677(2)
β/°	108.915(2)
γ/°	93.710(2)
Volume/Å^3	8159.2(6)
Z	4
ρ_{calc}g/cm^3	1.026
μ/mm^{-1}	0.475
F(000)	2664.0
Crystal size/mm^3	0.15 × 0.06 × 0.05
Radiation	Cu Kα (λ = 1.54178)
2Θ range for data collection/°	4.094 to 128.094

Index ranges	-21 ≤ h ≤ 21, -23 ≤ k ≤ 23, -26 ≤ l ≤ 26
Reflections collected	56825
Independent reflections	26618 [R_{int} = 0.0873, R_{sigma} = 0.1008]
Data/restraints/parameters	26618/66/1810
Goodness-of-fit on F^2	1.041
Final R indexes [I>=2σ (I)]	R_1 = 0.0962, wR_2 = 0.2763
Final R indexes [all data]	R_1 = 0.1333, wR_2 = 0.3066
Largest diff. peak/hole / e Å^{-3}	0.37/-0.48

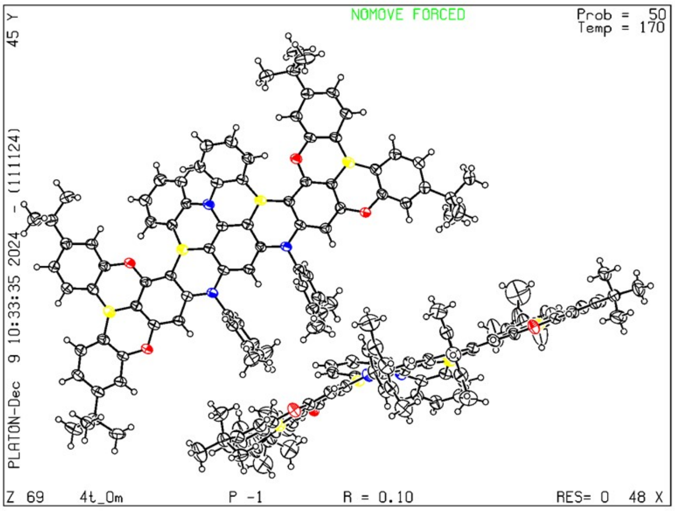

Figure S18. ORTEP of **4M-BOB4** with 50% thermal ellipsoids.

Table S2. Crystal data and structure refinement for 4F-BOB4.

Empirical formula	$C_{82}H_{65}B_4F_4N_3O_4$
Formula weight	1275.61
Temperature/K	150
Crystal system	monoclinic
Space group	$P2_1/c$
a/Å	21.1315(8)
b/Å	12.5670(5)
c/Å	32.2663(13)
α/°	90
β/°	91.372(3)
γ/°	90
Volume/Å^3	8566.2(6)
Z	4
*ρ*calcg/cm^3	0.989
μ/mm-1	0.528
F(000)	2664.0
Crystal size/mm^3	0.08 × 0.03 × 0.01
Radiation	Cu Kα (*λ* = 1.54178)
2*Θ* range for data collection/°	5.48 to 117.854

Index ranges	-23 ≤ h ≤ 23, -13 ≤ k ≤ 12, -33 ≤ l ≤ 35
Reflections collected	58324
Independent reflections	11915 [Rint = 0.0966, Rsigma = 0.0724]
Data/restraints/parameters	11915/0/887
Goodness-of-fit on F^2	1.157
Final R indexes [I>=2σ (I)]	R1 = 0.1153, wR2 = 0.3120
Final R indexes [all data]	R1 = 0.1562, wR2 = 0.3479
Largest diff. peak/hole / e Å^{-3}	0.38/-0.31

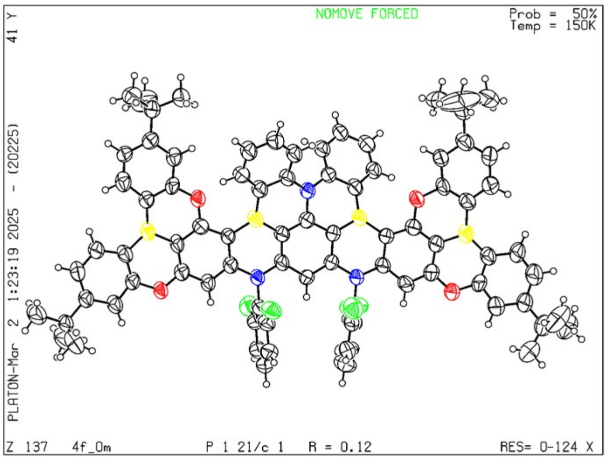

Figure S19. ORTEP of **4F-BOB4** with 50% thermal ellipsoids.

Table S3 Crystal data and structure refinement for 4M-BOB3.

Empirical formula	C87H82B3Cl2N3O4
Formula weight	1336.88
Temperature/K	170
Crystal system	monoclinic
Space group	P21
a/Å	13.9689(6)
b/Å	19.0091(8)
c/Å	14.0226(6)
α/°	90
β/°	103.179(3)
γ/°	90
Volume/Å^3	3625.4(3)
Z	2
ρcalcg/cm^3	1.225
μ/mm^{-1}	1.225
F(000)	1412.0
Crystal size/mm^3	0.08 × 0.04 × 0.03
Radiation	Cu Kα (*λ* = 1.54178)
2*Θ* range for data collection/°	6.474 to 117.862

Index ranges	-14 ≤ h ≤ 14, -21 ≤ k ≤ 20, -15 ≤ l ≤ 15
Reflections collected	19344
Independent reflections	9359 [Rint = 0.0768, Rsigma = 0.0947]
Data/restraints/parameters	9359/13/908
Goodness-of-fit on F^2	1.036
Final R indexes [I>=2σ (I)]	R1 = 0.0740, wR2 = 0.1843
Final R indexes [all data]	R1 = 0.0983, wR2 = 0.2003
Largest diff. peak/hole / e $Å^{-3}$	0.49/-0.61

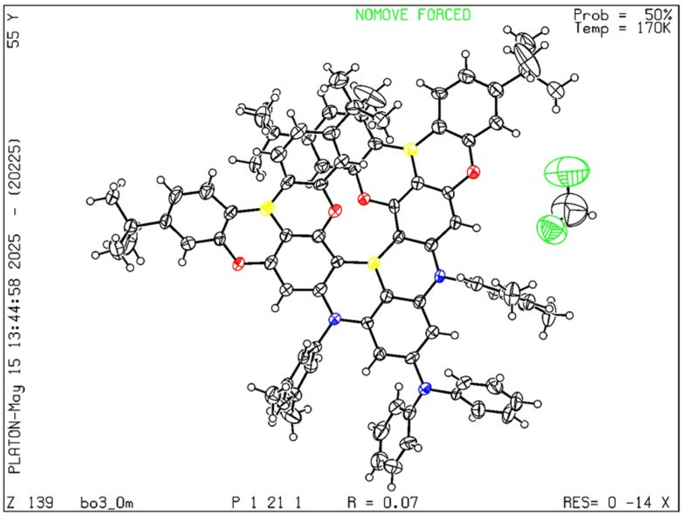

Figure S20. ORTEP of **4M-BOB3** with 50% thermal ellipsoids.

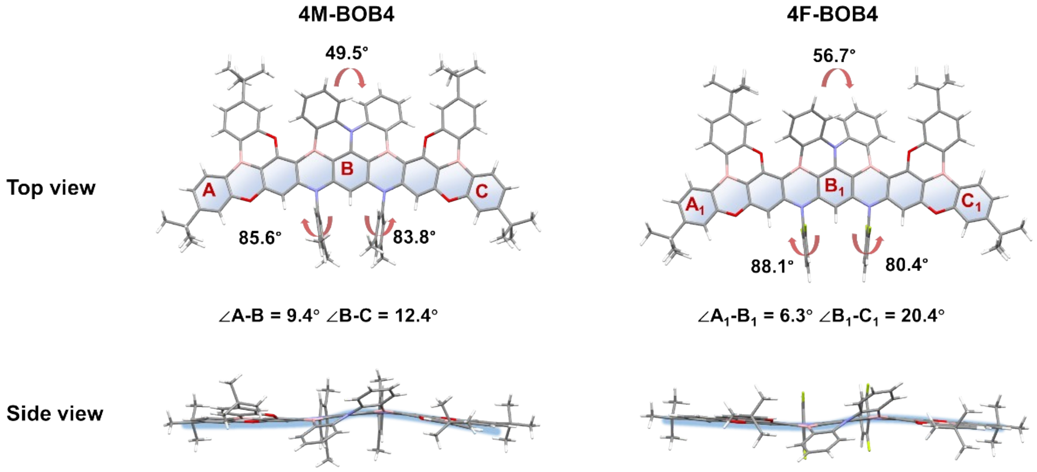

Figure S21. Single-crystal structure of the **4M-BOB4** and **4F-BOB4**, respectively.

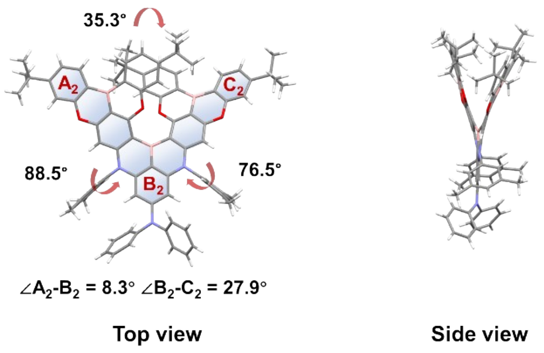

Figure S22. Single-crystal structure of the **4M-BOB3**.

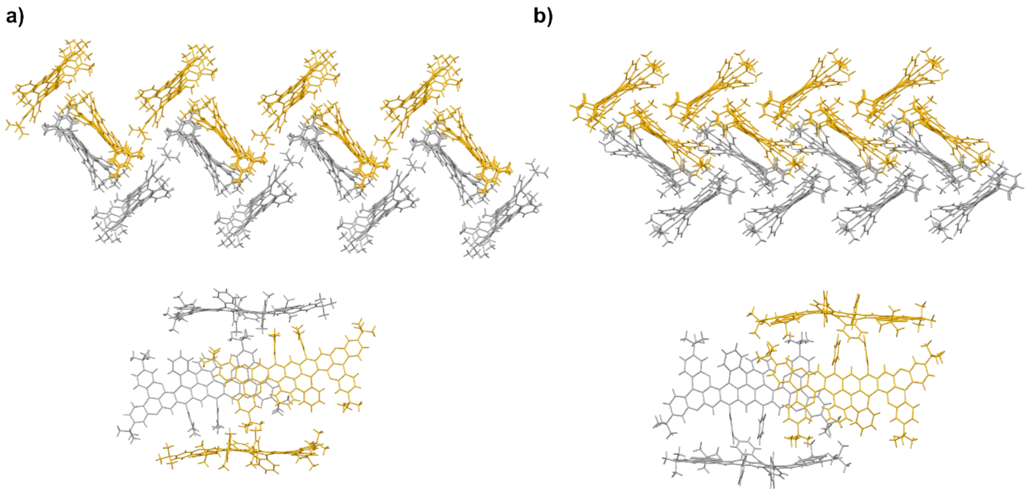

Figure S23. Single-crystal packing modes of the a) **4M-BOB4** and b) **4F-BOB4**, respectively.

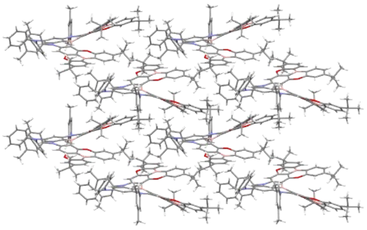

Figure S24. Single-crystal packing modes of the **4M-BOB3**.

4.Thermal and Electrochemical Characterization

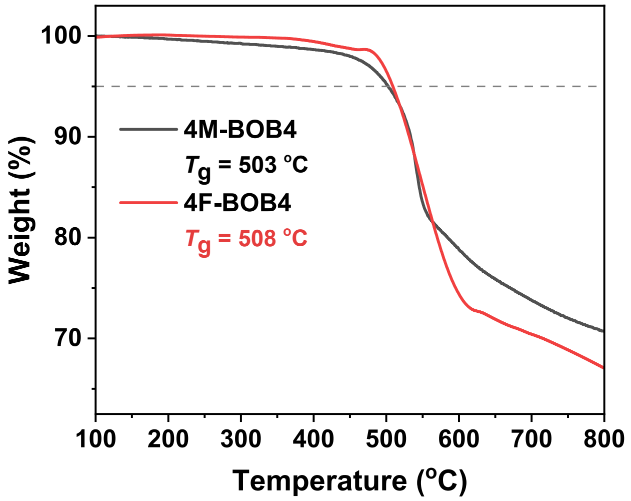

Figure S25. Thermogravimetric analysis (TGA) curve of **4M-BOB4** and **4F-BOB4**.

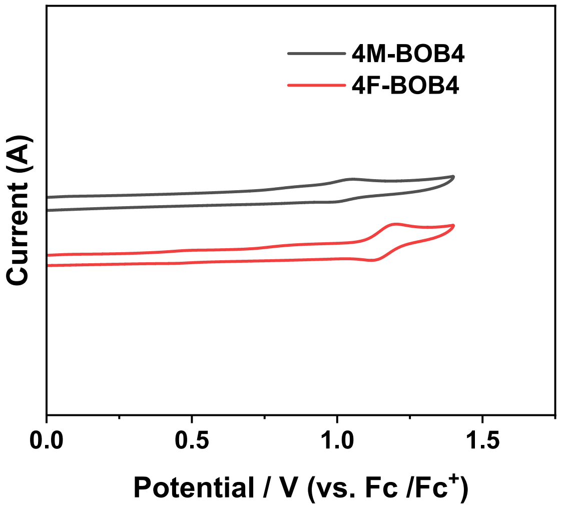

Figure S26. Cyclic voltammograms of **4M-BOB4** and **4F-BOB4** measured in dry DCM containing tetrabutylammonium hexafluorophosphate (Bu_4NPF_6, 0.1 M) as electrolyte and ferrocene/ferrocenium (Fc/Fc^+, 1.0×10^{-3} M) as standard.

Table S4. Electrochemical properties of 4M-BOB4 and 4F-BOB4.

Compound	E_g[a] [eV]	E_{ox}[b] [V]	HOMO[c] [eV]	LUMO[d] [eV]
4M-BOB4	2.74	0.94	-5.21	-2.47
4F-BOB4	2.79	1.09	-5.36	-2.57

[a] The optical band gap (E_g) were estimated from the tangent drawn to the onset of the absorption spectrum; [b] Oxidation potential determined by electrochemical measurements [c] HOMO levels were measured from the oxidation potential by cyclic voltammetry with ferrocene as the external standard; [d] LUMO energy levels were calculated from HOMO and optical bandgap (E_g);

5. Computational Methods

The calculations were performed with the Gaussian 16 package. [S2] S_0 states were carried out using DFT and hole-electron distribution of S_1, T_1, T_2 and T_3 states. The SOC matrix elements using time-dependent density functional theory (TD-DFT) with the dispersion corrected PBE0 functional and def2-SVP basis set. [S3] The topological properties and electrostatic potential on van der Waals surface and analysis were determined with the Multiwfn program and VMD software packages. [S4] The SOC matrix elements were predicted by the PySOC program. [S5] The RMSDs of the optimized structures at S_0 and S_1 states were analyzed by VMD software. [S6] The Huang-Rhys (HR) factors and reorganization energies were calculated using the FCclasses software. [S7] The nucleusindependent chemical shift (NICS) calculation was done at GIAO-PBE0/def2-SVP level of theory. Bq atoms were inserted at the calculated positions and the Bq positions that are at the 1 Å away above the molecule were fixed with the assistant of Multiwfn 3.8 software, as well as the generation of isotropic chemical shielding surfaces (ICSS) and related quantities. [S8]

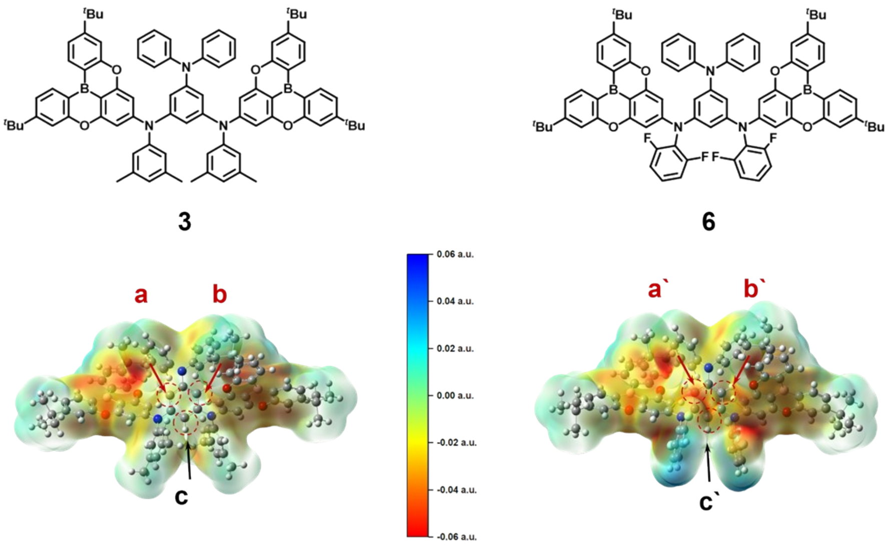

Figure S27. The electrostatic potential (ESP) of intermediates **3** and **6**.

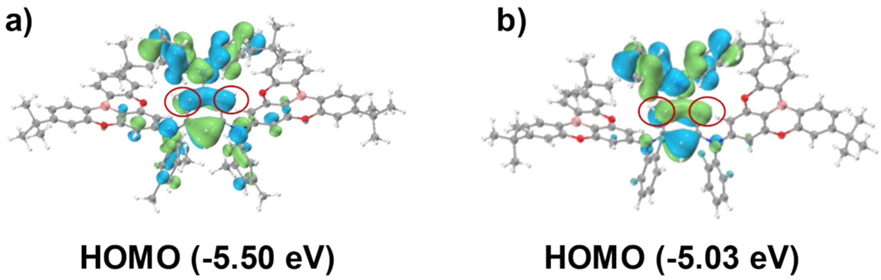

Figure S28. Molecular structure HOMO distributions of a) intermediates **3** and b) **6**.

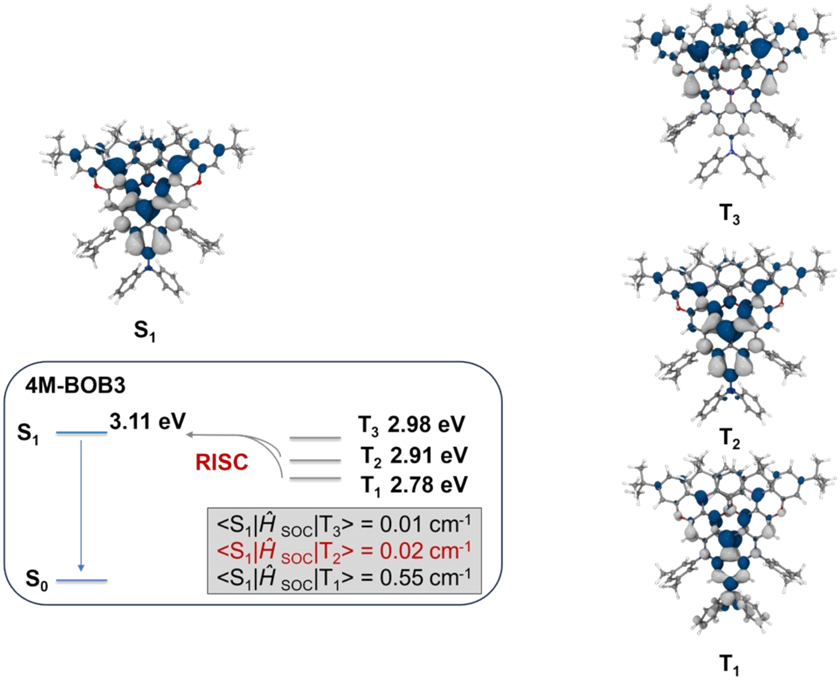

Figure S29. The hole(white), electron (blue) distributions and the energy level alignment and spin orbital coupling matrix elements (SOCME) of the excited states (S_1, T_1, T_2 and T_3) of **4M-BOB3**.

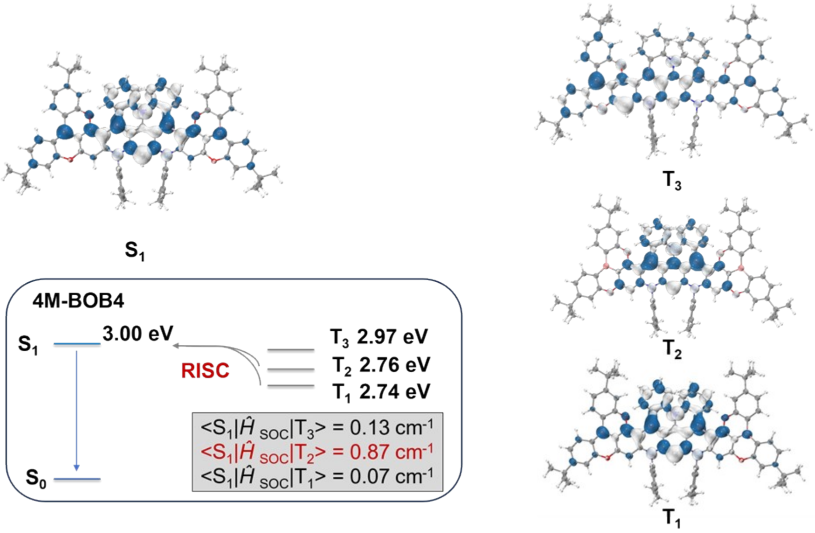

Figure S30. The hole(white), electron (blue) distributions and the energy level alignment and spin orbital coupling matrix elements (SOCME) of the excited states (S_1, T_1, T_2 and T_3) of **4M-BOB4**.

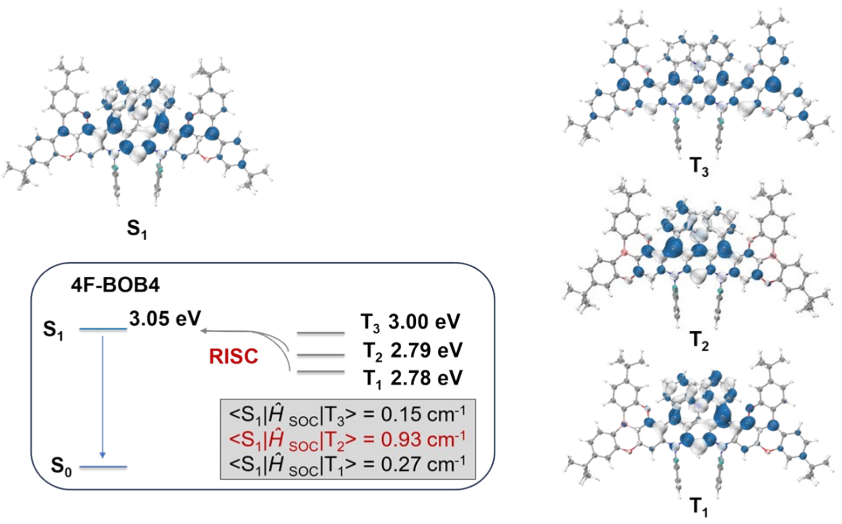

Figure S31. The hole(white), electron (blue) distributions and the energy level alignment and spin orbital coupling matrix elements (SOCME) of the excited states (S_1, T_1, T_2 and T_3) of **4F-BOB4**.

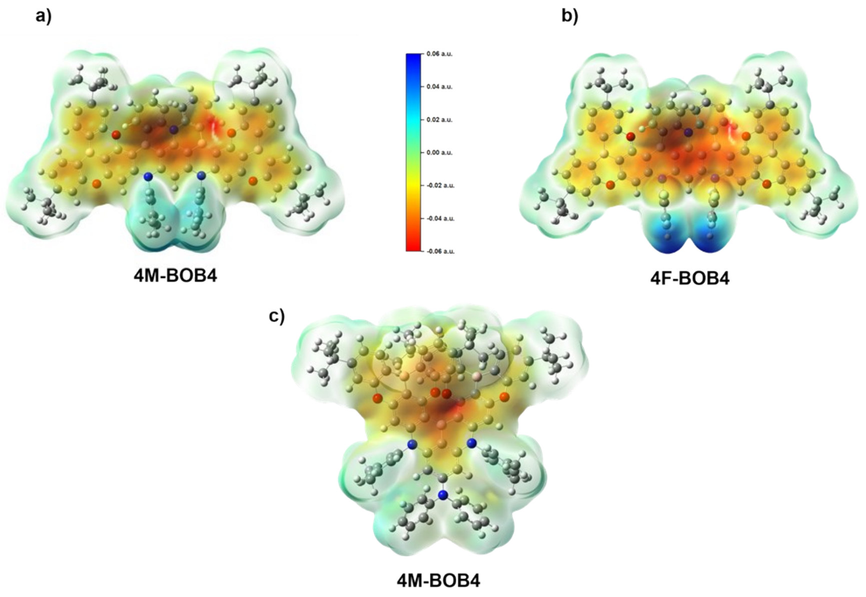

Figure S32. The electrostatic potential (ESP) of **4M-BOB4**, **4F-BOB4** and **4M-BOB3**.

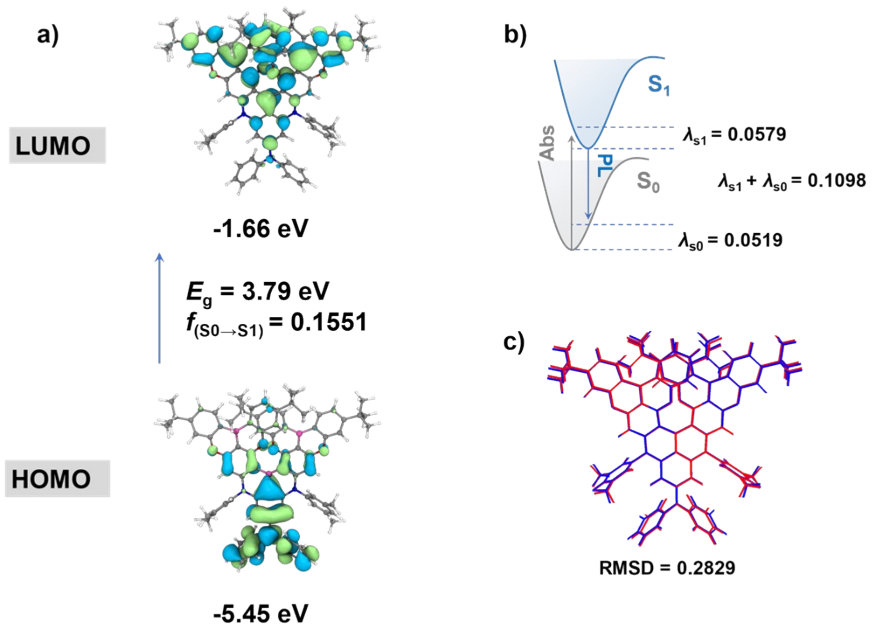

Figure S33. a) The HOMO and LUMO distributions and energy level diagrams of **4M-BOB3**; b) Calculated reorganization energy (λ) and c) root-mean-square-deviation values of **4M-BOB3**.

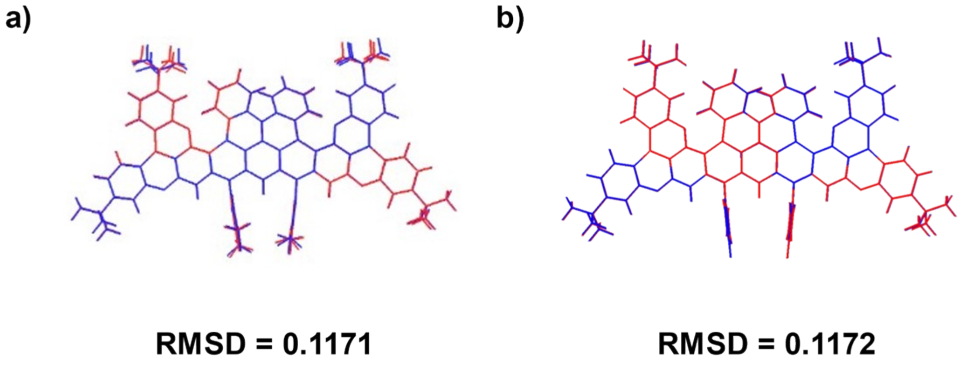

Figure S34. Configuration of S_1 (blue) and S_0 (red) and the RMSD values for **4M-BOB4** and **4F-BOB4**.

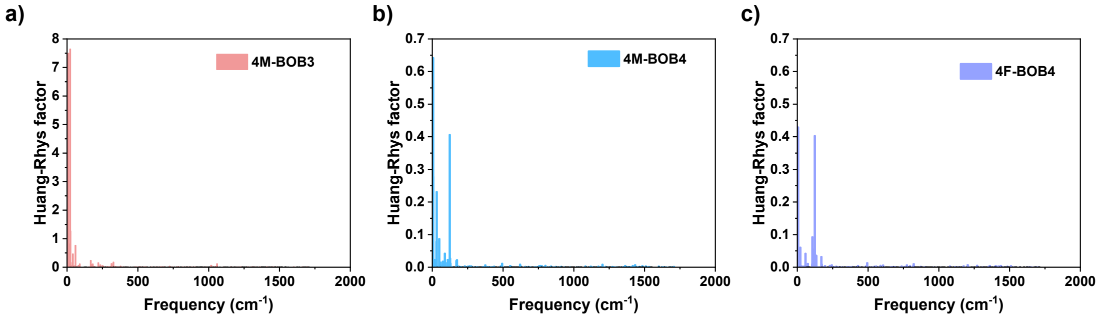

Figure S35. Huang-Rhys (HR) factor for the $S_1 \rightarrow S_0$ transition of **4M-BOB3**, **4M-BOB3** and **4F-BOB4**.

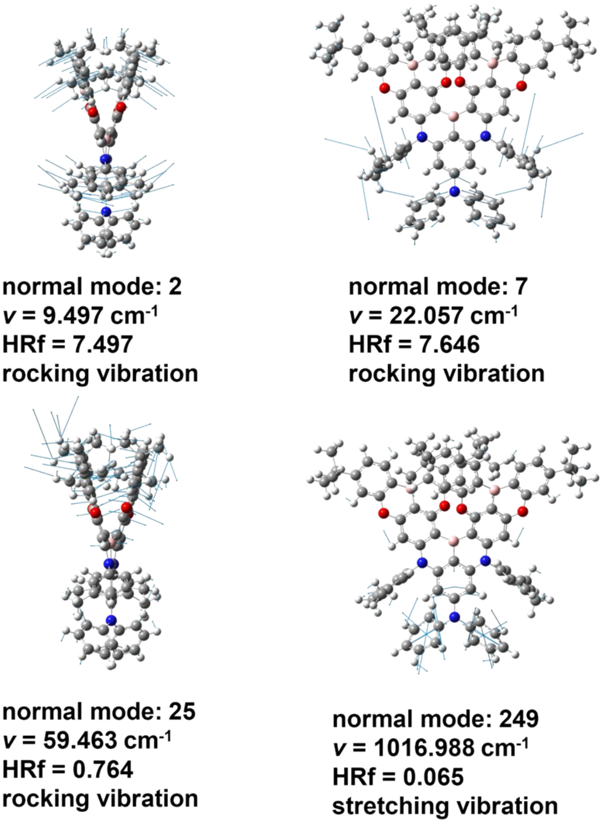

Figure S36. Representative normal modes of **4M-BOB4** from S_1 to S_0 transition.

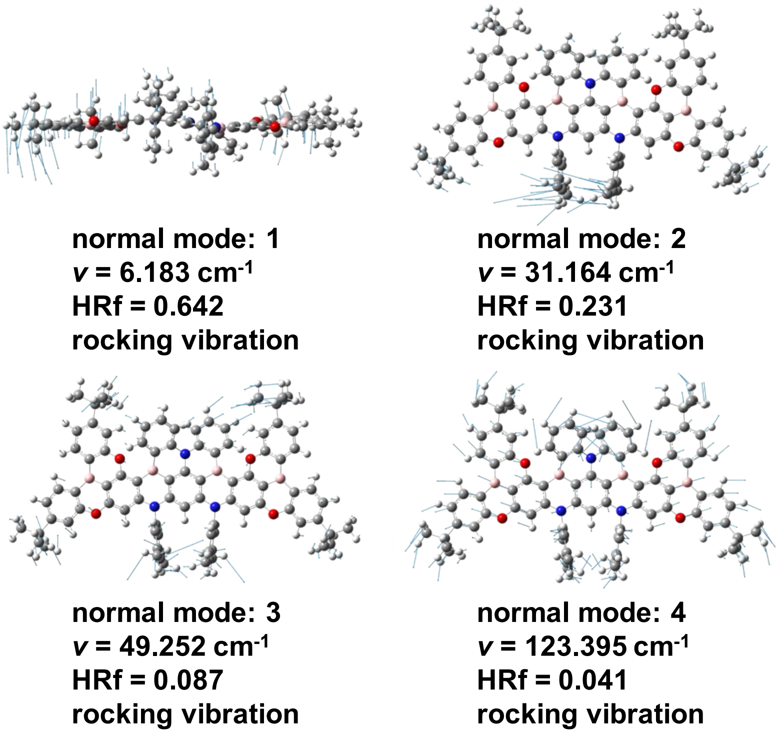

Figure S37. Representative normal modes of **4M-BOB4** from S_1 to S_0 transition.

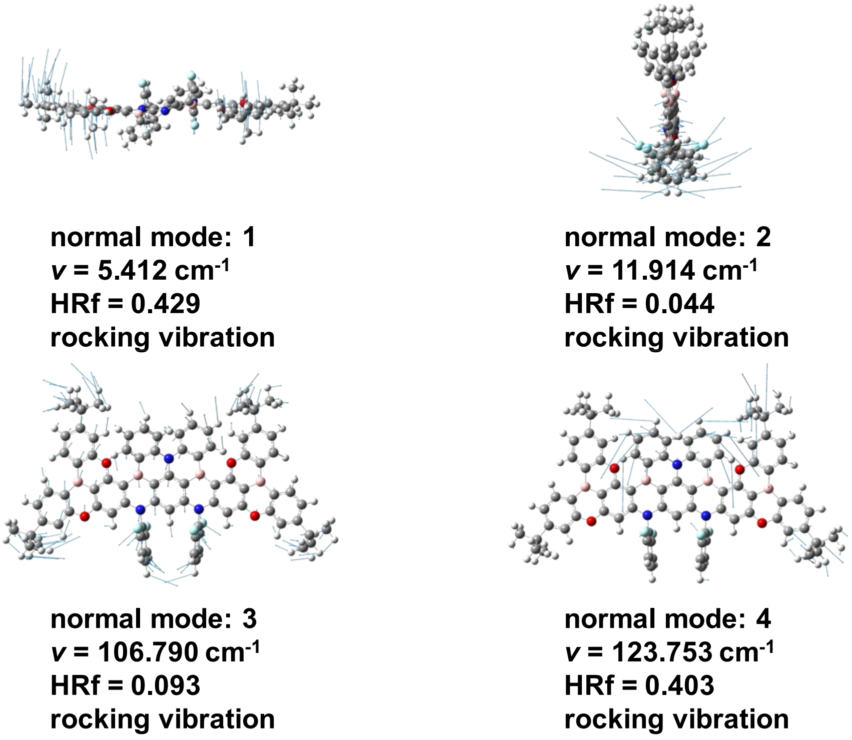

Figure S38. Representative normal modes of **4F-BOB4** from S_1 to S_0 transition.

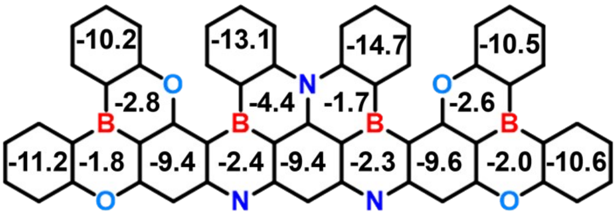

Figure S39. DFT-calculated NICS(1)$_{zz}$ values of the luminescent core.

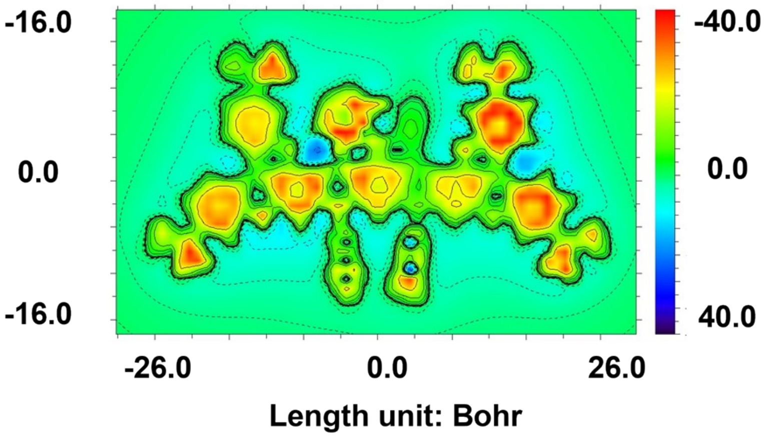

Figure S40. 2D ICSS map of **4M-BOB4** at 1Å above the XY plane.

Table S5. Summary of TD-DFT Calculation for 4M-BOB4 and 4F-BOB4 at the PBE0/def2-SVP level of theory.

compound	S_n	Mult	eV	nm	f	orb
4M-BOB4	1	Singlet	2.9996	413.33	0.9109	H-L:0.894;H-L3:0.061
	2	Singlet	3.2379	382.91	0.001	H-L2:0.608;H1-L:0.299
	3	Singlet	3.3724	367.64	0.0382	H-L1:0.816;H-L2:0.061
4F-BOB4	1	Singlet	3.0468	406.93	0.8181	H-L:0.882;H-L3:0.073
	2	Singlet	3.2946	376.33	0.0012	H-L2:0.586;H1-L:0.289
	3	Singlet	3.4177	362.77	0.2249	H-L1:0.715;H1-L:0.128

6. Photophysical properties

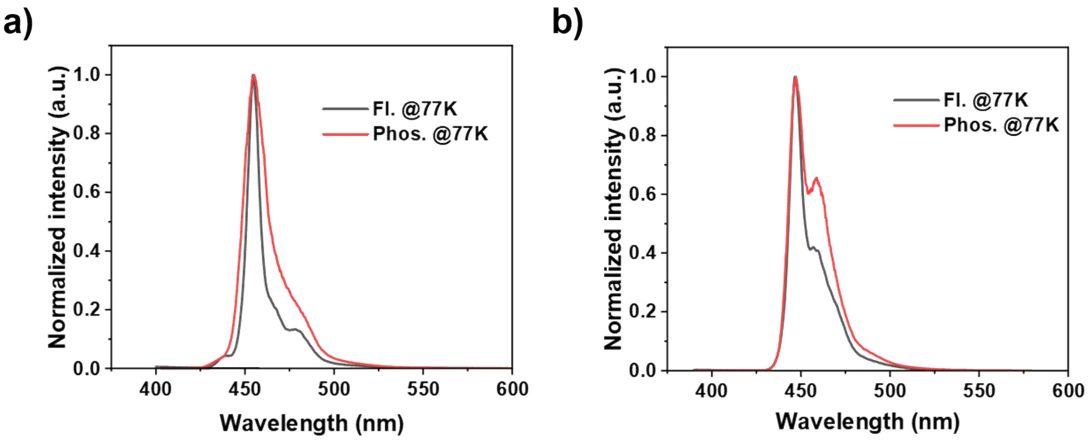

Figure S41. Fluorescence and phosphorescence spectra (77 K) of a) 4M-BOB4 and b) 4F-BOB4 in toluene solutions at 1×10^{-5} M

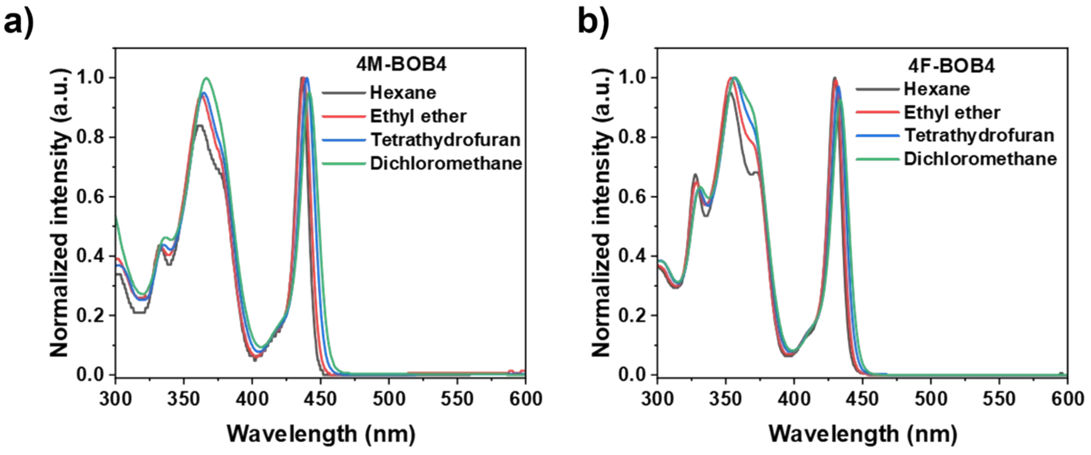

Figure S42. UV-vis absorption spectra of a) 4M-BOB4 and b) 4F-BOB4 were measured in different solvents

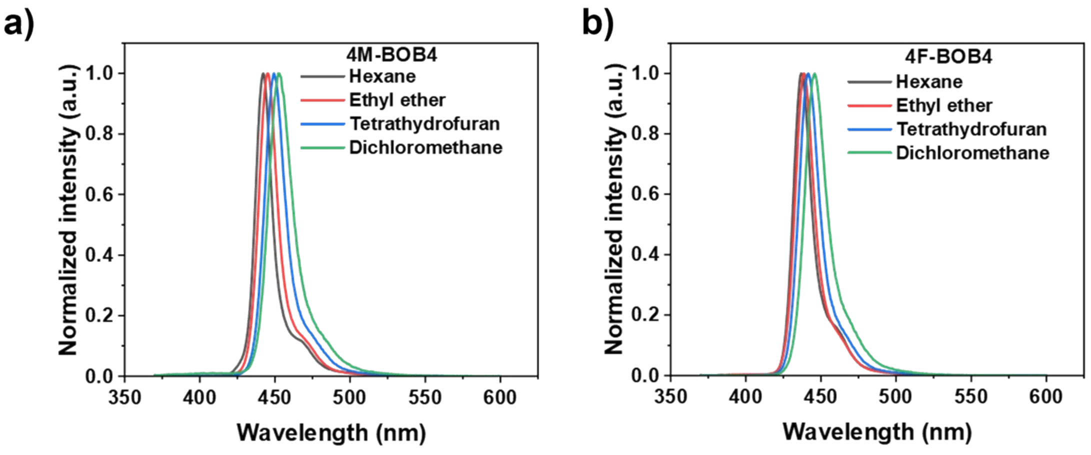

Figure S43. PL spectra of a) 4M-BOB4 and b) 4F-BOB4 were measured in different solvents.

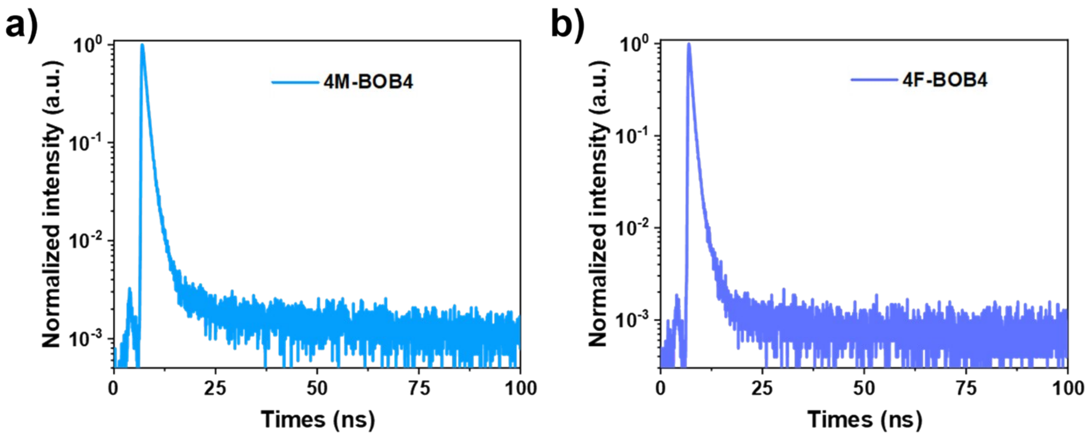

Figure S44. Transient photoluminescence curves of 4M-BOB4 and 4F-BOB4 in DOBNA-OAr (5 wt% doped film).

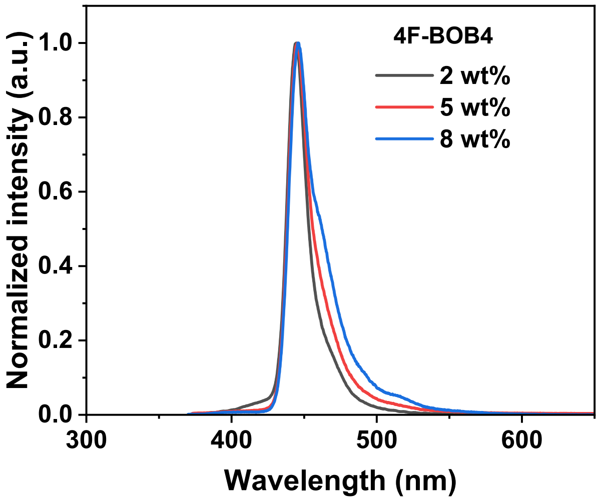

Figure S45. PL spectra of 4F-BOB4 doped in DOBNA-OAr films at 2, 5, and 8 wt%.

Table S6. Summary of the photophysical properties of 4F-BOB4 doped in DOBNA-OAr films at 2, 5, and 8 wt%.

compound	Doping ratio (wt%)	PL (nm)	FWHM (nm)
4F-BOB4	2	444	16
	5	445	18
	8	445	23

Table S7. Summary of photophysical properties for 4M-BOB4 and 4F-BOB4.

Compound	τ_{PF}/τ_{DF} ns/µs	ks r ×10^7 s^{-1}	ks nr ×10^6 s^{-1}	k_{ISC} ×10^8 s^{-1}	k_{RISC} ×10^6 s^{-1}
4M-BOB4	5.82/2.57	2.61	4.60	1.39	2.22
4F-BOB4	5.53/4.85	1.92	4.22	1.56	1.59

$$k_p = \frac{1}{\tau_{PF}}$$

$$k_d = \frac{1}{\tau_{DF}}$$

$$k_r^S = k_p \phi_{PF}$$

$$k_{nr}^S = k_p \frac{\phi_{PF}}{\phi_{\mathbf{all}}}(1 - \phi_{\mathbf{all}})$$

$$k_{ISC} = k_p \frac{\phi_{DF}}{\phi_{\mathbf{all}}} - k_d \frac{\phi_{DF}}{\phi_{PF}}$$

$$k_{RISC} = k_d \frac{\phi_{\mathbf{all}}}{\phi_{PF}}$$

where k_p, and k_d are the radiative decay rate for prompt and delayed fluorescence, respectively; Φ_{all} is the total photoluminescence quantum efficiency; ks rand ks nr, are the radiative and nonradiative decay rate constants from a singlet excited state, respectively, k_{ISC} and k_{RISC} are the intersystem crossing and reverse intersystem crossing rate constants respectively. Thus, the quantum efficiencies of prompt (Φ_{PF}) and delayed emission (Φ_{DF}) are evaluated by the corrected estimation method and the rate constants were calculated according to the reported method. [S9]

7. Device Fabrication and Measurement

HATCN, TAPC, TCTA, mCBP, TmPyPB and Liq were obtained from commercial sources. OLED devices employing 4F-BOB4 as an emitter with the following structure were fabricated: ITO/HAT-CN (10 nm)/TAPC (35 nm)/TCTA (10 nm)/mCBP (10 nm)/ DOBNA-OAr: x= (2, 5, 8) wt% emitters/ TmPyPB (40 nm)/Liq (3 nm)/Al (100 nm), where DOBNA-OAr was synthesized according to literature reports. All the functional layers were fabricated on pretreated ITO substrates. ITO glass substrates were cleaned by ultrasonication with detergent, alcohol, acetone, and deionized water and then dried at 100 °C in an oven for more than 60 mins. The EL spectra, current density-voltage-luminance (*J*-*V*-*L*) characteristics and external quantum efficiency were measured with a Keithley source measurement unit (Keithley 2400) and quantum efficiency integrating sphere system (XP-FOIS). Notably, all the characterizations of the devices were carried out at room temperature under ambient conditions.

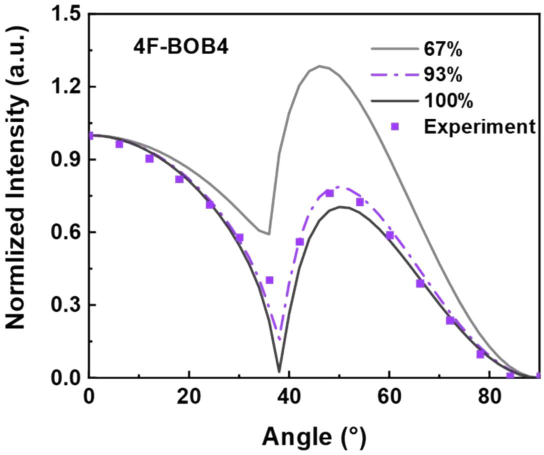

Figure S45. Angle-dependent p-polarized PL intensity and simulation curve for the emitting layer 4F-BOB4 with the 5wt % doping ratios.

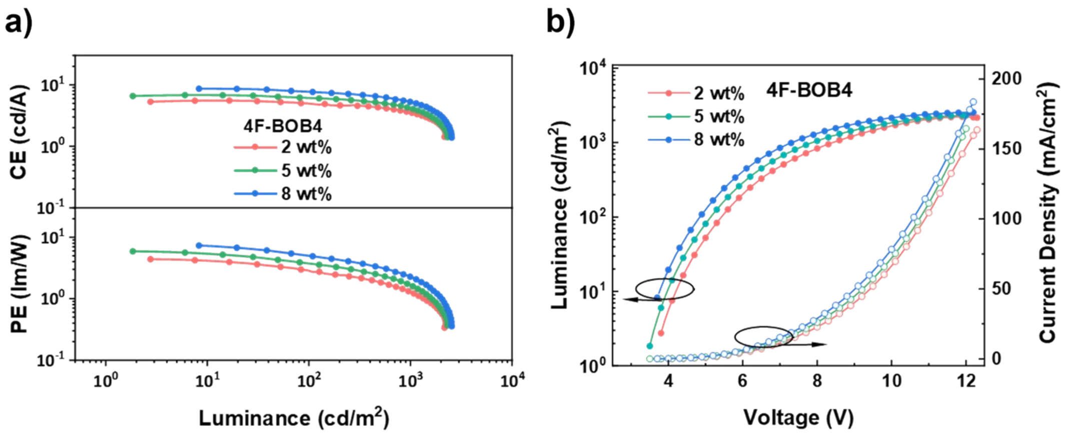

Figure S46. Characteristics of OLED device using 4F-BOB4 as an emitter. a) Current efficiency and power efficiency versus luminance curves. b) Current density-voltage-luminance characteristics.

Table S8. Summary of the representative deep-blue OLEDs performance employing MR-TADF emitters ($CIE_y \leq 0.055$).

**Emitter**	**Host**	**λ_{EL} [nm]**	**FWHM [nm]**	**$EQE_{max/100/1000}$ [%]**	**CIE (x, y)**	**Ref.**
DMACN-B	DPEPO	444	44	10.0/-/-	(0.151, 0.045)	*Adv. Funct. Mater*. **2021**, 31, 2009488
B-O-dpa	DPEPO	443	32	16.3/6.5/2.2	(0.15, 0.05)	*ACS Appl. Mater. Interfaces*. **2021**, 13, 45798
tPBisICz	mCP: TSPO1	452	21	23.1/-/-	(0.15, 0.05)	*Adv. Sci.* **2021**, 8, 2101137.
BIC-mCz	PPF	432	42	19.4/-/-	(0.16, 0.05)	*Angew. Chem.* **2022**, 134, e202206916
mDBIC		431	42	13.5/-/-	(0.16, 0.05)	
BOBO-Z	mCBP	445	18	13.6/9.8/3.3	(0.15, 0.04)	*Adv. Mater*. **2022,** 34, 2107951
2B-DTACrs	mCP	447	26	14.8/-/-	(0.150, 0.044)	*Chem. Commun.* **2022**, 58, 9377
CzBO	mCBP	448	30	13.4/8.4/3.5	(0.15, 0.05)	*Angew. Chem. Int. Ed.* **2022**, 61, e202205684
TDBA-PAS	DPEPO	435	50	22.3/17.9/-	(0.155, 0.042)	*Adv. Mater*. **2022**, 34, 2200537
tDIDCz	DPEPO	401	17	2.5/-/-	(0.164, 0.055)	*Adv. Optical Mater*. **2023**, 11, 2202176.
*γ*m-ICz		412	36	3.0/-/-	(0.160, 0.034)	
o-ICz		433	40	3.0/-/-	(0.165, 0.046)	
CzCO	PPF	432	35	15.6/6.0/5.9	(0.154, 0.047)	*Angew. Chem. Int. Ed.* **2023**, 135, e202215226
t-mp3ICz	mCP: TSPO1	446	21	24.3/4.4/-	(0.154, 0.044)	*Materials Today* **2023**, 69, 88-96
NOBN	TSPO1	409	37	8.5/-/-	(0.173, 0.055)	*Angew. Chem. Int. Ed.* **2023**, 62, e202215522
MesB-DIDOBNA-N	TSPO1	402	21	16.2/3.5/-	(0.170, 0.049)	*Adv. Mater*. **2023**, 35, 2300997
DBCz-Mes	mCBP	452	17	33.9/-/-	(0.144, 0.058)	*Sci. Adv*. **2023**, 9, eadh1434
DOB2-DABNA-A	DOBNA-Tol	452	24	24.1/23.3/21.6	(0.145, 0.049)	*Nat Commun.* **2024**, 15, 2361
f-DOABNA	DOBNA-Tol	445	28	19.9/12.9/6.7	(0.153, 0.056)	*Adv. Mater*. **2024**, 36, 2402289
DB	DOBNA-OAr	443	26	23.4/17.4/9.0	(0.154, 0.048)	*Adv. Mater*. **2024**, 36, 2308314.
DB-O		445	24	27.5/21.3/11.4	(0.150, 0.041)	
DB-S		447	24	29.3/23.6/12.2	(0.148, 0.047)	

[B-N]N2	SiCzCz	441	20	20.3/-/-	(0.152, 0.046)	*Adv. Mater.* **2024**, 36, 2409706
DOBN	mCP	449	20	35.4/26.2/-	(0.15, 0.04)	*ACS Materials Lett.* **2024**, 6, 3246-3253
5Cz-BO	TSPO1	416	36	22.8/-/-	(0.163, 0.046)	*Adv. Mater*. **2024**, 36, 2313602
Py-BN	DOBNA-OAr	444	21	15.8/7.4/3.9	(0.153, 0.045)	*Angew. Chem. Int. Ed.* **2024**, 63, e202408522
DPA-B2	SiTrzCz2	442	30	21.9/15.6/10.0	(0.150, 0.052)	*Nat. Photon*. **2024**, 18, 1161
DPA-B3	SiTrzCz2	448	15	30.6/25.8/17.2	(0.150, 0.050)	
DPA-B4	SiTrzCz2	456	13	30.4/28.3/23.3	(0.140, 0.050)	
DBNO	SiTrzCz2	448	26	22.7/12.2/3.1	(0.148, 0.047)	*Chem. Sci*. **2025**
DMBNO		449	25	32.3/20.7/5.5	(0.148, 0.046)	
2F-PAB	PPF	436	35	4.2/-/-	(0.157, 0.044)	*Adv. Funct. Mater*. **2025**, 35, 2419679
Me-PAB		448	29	19.8/-/-	(0.146, 0.046)	
3B-DPA	DBFPO	440	14	18.7/6.7/-	(0.154, 0.031)	*Angew. Chem. Int. Ed*. **2025**, 64, e202503320
2	mCBP	442	27	11.0/-/-	(0.153, 0.036)	*Angew. Chem. Int. Ed*. **2025**, 137 e202510891
3		441	25	9.1/-/-	(0.155, 0.030)	
4		441	25	8.1/-/-	(0.155, 0.030)	
5		451	26	9.3/-/-	(0.146, 0.053)	
ICzF	mCP: TSPO1	440	21	11.3/-/-	(0.169, 0.054)	*Adv. Sci.* **2025**, e04625
BN-CP	PPF	432	32	23.3/-/-	(0.16, 0.04)	*Natl. Sci. Rev.,* 12, **2025**, nwaf250
3BON	SiTrzCz2	441	19	23.8/20.1/13.7	(0.158, 0.0402)	*Angew. Chem. Int. Ed.* **2025**, e202518651
**4F-BOB4**	**DOBNA-OAr**	**443**	**17**	**20.9/18.8/12.8**	**(0.156, 0.030)**	**This work**

8. Reference

[S1] R. W. Weerasinghe, S. Madayanad Suresh, D. Hall, T. Matulaitis, A. M. Z. Slawin, S. Warriner, Y.-T. Lee, C.-Y. Chan, Y. Tsuchiya, E. Zysman-Colman, C. Adachi, A Boron, Nitrogen, and Oxygen Doped π-Extended Helical Pure Blue Multiresonant Thermally Activated Delayed Fluorescent Emitter for Organic Light Emitting Diodes That Shows Fast kRISC Without the Use of Heavy Atoms. *Adv. Mater*. **2024**, 36, 2402289.

[S2] M. J. Frisch, G. W. T., H. B. Schlegel, G. E. Scuseria, ; M. A. Robb, J. R. C., G. Scalmani, V. Barone, ; G. A. Petersson, H. N., X. Li, M. Caricato, A. V. Marenich, ; J. Bloino, B. G. J., R. Gomperts, B. Mennucci, H. P. Hratchian, ; J. V. Ortiz, A. F. I., J. L. Sonnenberg, D. WilliamsYoung, ; F. Ding, F. L., F. Egidi, J. Goings, B. Peng, A. Petrone, ; T. Henderson, D. R., V. G. Zakrzewski, J. Gao, N. Rega, G. Zheng, W. L., M. Hada, M. Ehara, K. Toyota, R. Fukuda, ; J. Hasegawa, M. I., T. Nakajima, Y. Honda, O. Kitao, H. Nakai, T. Vreven, K. T., J. A. Montgomery, Jr., J. E. Peralta, F. Ogliaro, M. J. B., J. J. Heyd, E. N. Brothers, K. N. Kudin, V. N. Staroverov, T. A. K., R. Kobayashi, J. Normand, K. Raghavachari, A. P. R., J. C. Burant, S. S. Iyengar, J. Tomasi, M. C., J. M. Millam, M. Klene, C. Adamo, R. Cammi, J. W. Ochterski, R. L. M., K. Morokuma, O. Farkas, J. B. Foresman, D. J. Fox, Wallingford, CT, **2016**.

[S3] L. Kronik, T. Stein, S. Refaely-Abramson, R. Baer, *J. Chem. Theory Comput.* **2012**, 8, 1515.

[S4] a) T. Lu, F. Chen, *J. Comput. Chem*. **2012**, *33*, 580-592; b) W. Humphrey, A. Dalke, K. Schulten, *J. Mol. Graph. Moedl.* **1996**, 14, 33.

[S5] a) X. Gao, S. Bai, D. Fazzi, T. Niehaus, M. Barbatti, W. Thiel, *J. Chem. Theory Comput.* **2017**, *13*, 515-524; b) Z. Shuai, *Chin. J. Chem.* **2020**, *38*, 1223-1232.

[S6] Humphrey, W.; Dalke, A.; Schulten, K., VMD: Visual molecular dynamics. *Journal of Molecular Graphics*. **1996**, 14, 33-38.

[S7] a) J. Cerezo, F. Santoro, *J. Comput. Chem*. 2023, 44, 626; b) Huang, K.; Rhys, A.; Mott, N. F., Theory of light absorption and non-radiative transitions in *F*-centres Series A. *Mathematical and Physical Sciences*. **1950**, 204, 406-423.

[S8] (a) T. Lu, F. Chen, J. Comput. *Chem*. **2012**, 33, 580-592; (b) H. Fallah-Bagher-Shaidaei, C. S. Wannere, C. Corminboeuf, R. Puchta, P. v. R. Schleyer, *Org. Lett*. **2006**, 8, 863-866; (c) S. Klod, E. Kleinpeter, *J. Chem. Soc*., *Perkin Trans*. **2001**, 1893-1898; (d) Z. Liu, T. Lu, Q. Chen, *Carbon*. **2020**, 165, 468-475.

[S9] Y. Tsuchiya, S. Diesing, F. Bencheikh, Y. Wada, P. L. dos Santos, H. Kaji, E. Zysman-Colman, I. D. W. Samuel, C. Adachi, *J. Phys. Chem. A* **2021**, *125*, 8074-8089.
